# Nuclear bodies formed by polyQ-ataxin-1 protein are liquid RNA/protein droplets with tunable dynamics

**DOI:** 10.1038/s41598-020-57994-9

**Published:** 2020-01-31

**Authors:** Sunyuan Zhang, Elizabeth Hinde, Molly Parkyn Schneider, David A. Jans, Marie A. Bogoyevitch

**Affiliations:** 1 Department of Biochemistry and Molecular Biology, University of Melbourne, Parkville, Victoria 3010 Australia; 2 Bio21 Molecular Science and Biotechnology Institute, University of Melbourne, Parkville, Victoria 3010 Australia; 3 School of Physics, University of Melbourne, Parkville, Victoria, 3010 Australia; 4 Nuclear Signalling Lab., Department of Biochemistry and Molecular Biology, Monash University, Clayton, Victoria 3800 Australia

**Keywords:** Imaging, Nuclear organization

## Abstract

A mutant form of the ataxin-1 protein with an expanded polyglutamine (polyQ) tract is the underlying cause of the inherited neurodegenerative disease spinocerebellar ataxia 1 (SCA1). In probing the biophysical features of the nuclear bodies (NBs) formed by polyQ-ataxin-1, we defined ataxin-1 NBs as spherical liquid protein/RNA droplets capable of rapid fusion. We observed dynamic exchange of the ataxin-1 protein into these NBs; notably, cell exposure to a pro-oxidant stress could trigger a transition to slower ataxin-1 exchange, typical of a hydrogel state, which no longer showed the same dependence on RNA or sensitivity to 1,6-hexanediol. Furthermore, we could alter ataxin-1 exchange dynamics either through modulating intracellular ATP levels, RNA helicase inhibition, or siRNA-mediated depletion of select RNA helicases. Collectively, these findings reveal the tunable dynamics of the liquid RNA/protein droplets formed by polyQ-ataxin-1.

## Introduction

Within eukaryotic cells, complex biochemical reactions are facilitated by the concentration and restriction of key enzymes, substrates and regulators within well-defined membrane-bound organelles. In addition, numerous membrane-less intracellular compartments are important for a range of essential activities in both the cytoplasm (e.g. stress granules storing RNA molecules from stalled translation in response to environmental stresses^1^) and the nucleus (e.g. nucleoli functioning in the biogenesis of ribosome subunits^2^, Cajal bodies in the processing and modification of short non-coding RNAs^3^, or promyelocytic leukemia (PML) bodies acting as sumoylation factories in response to interferon and oxidative stress^4^). The formation of many of these membrane-less organelles is now understood to proceed via a phase separation process of specific constituent proteins, RNA and/or DNA molecules^5^. Thus, after a certain critical concentration threshold is exceeded, molecular assemblies of these constituents are formed with liquid-like behaviors that include fusing ability, viscous fluid dynamics, and high exchange rates with their surroundings in the nucleoplasm or cytoplasm^6–10^. This process of protein phase separation is now viewed as an essential mechanism for efficient compartmentalization that can be rapidly responsive to environmental challenges or intracellular changes^11,12^.

Proteins that can undergo phase separation usually contain sequences conforming to either a low complexity region (LCR) or prion-like domain (PrLD)^8,11,13^; these are protein domains typically with low amino acid diversity and little conformational heterogeneity^5,11^. These disordered structural characteristics can also contribute to an additional change known as protein phase transition, in which liquid-like condensates continue to become less dynamic and so form a more viscoelastic hydrogel or solid-like fibrous aggregates^12,14^. Many factors can promote the phase transition process such as time, pH, and altered amino acid sequences arising from gene mutations^8,9,15,16^. Thus, sequences typically conforming to LCR or PrLD motifs have been proposed as indicators of a protein’s propensity to undergo protein phase separation and phase transition^17,18^.

In addition to this importance of membrane-less organelle formation as part of normal cellular physiology, many mutant proteins linked particularly to neurodegenerative diseases have also been identified with LCR or PrLD motifs. Notable examples include the liquid-like condensates and further phase transition into hydrogel by amyotrophic lateral sclerosis (ALS) mutants of FUS or hnRNPA1^9,19,20^. In assessing the processes of phase separation and phase transition for other neurodegenerative diseases, it is notable that trinucleotide repeat expansions can be considered as a type of LCR or PrLD^17,21,22^. In spinocerebellar ataxia 1 (SCA1) patients, the mutant *ATXN1* gene contains CAG repeats that encode an expanded polyglutamine (polyQ) region, and the formation of distinct nuclear “inclusions” of these polyQ-ataxin-1 proteins in SCA1 patients and transgenic mice^23–25^. Initially these inclusions may lack the fibrillar structure typical of disease-causing amyloids^22,26^ but instead show highly dynamic exchange^27^. The main aim of our study is to provide the first comprehensive evaluation of the physical properties of these NBs, to allow us to define a relationship between NB dynamic exchange and toxicity. Here, we implement a suite of microscopy and biochemical approaches to define the nuclear bodies (NBs) formed by polyQ-ataxin-1 as dynamic liquid protein/RNA droplets. These NBs exhibit ready-to-fuse ability and high dynamics revealed by fluorescence fluctuation spectroscopy (FFS) and fluorescence recovery after photobleaching (FRAP). More importantly, we have observed the tunable dynamics of these ataxin-1 NBs, with their high dynamic liquid phase maintained by ATP and RNA helicases, and their low dynamic hydrogel phase triggered by environmental stress. Thus, models that explain the protein aggregation process and pathogenesis mechanism in SCA1 neurodegeneration should now be extended to include polyQ-ataxin-1 protein phase separation and transition.

## Results

### PolyQ-ataxin-1 phase separates into liquid droplets in cells

**Figure 1:**
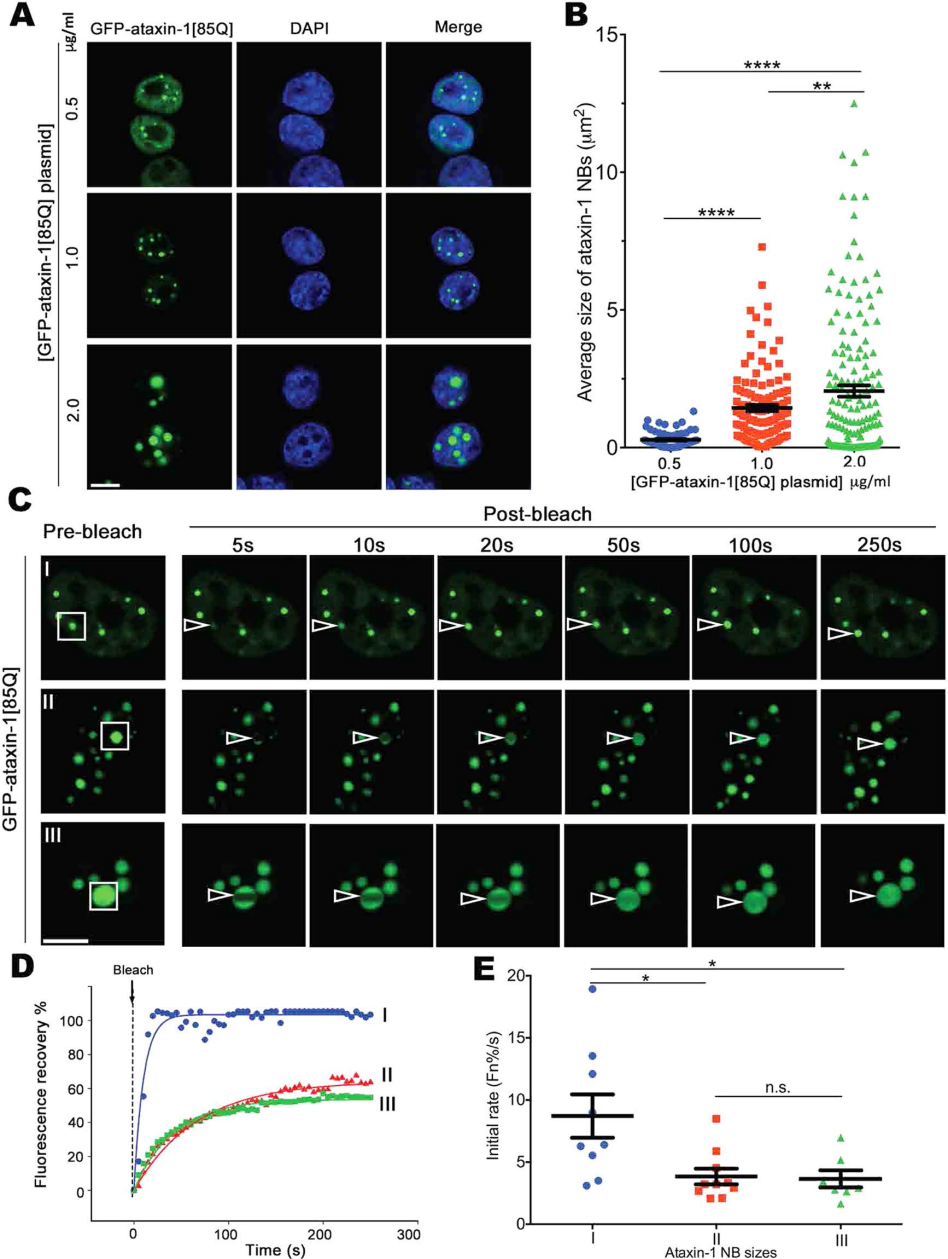
Ataxin-1 forms concentration-dependent nuclear bodies (NBs) that are highly dynamic. Neuro-2a cells were transfected to express GFP-ataxin-1[85Q]. (**A**) At 24 h post-transfection with different plasmid concentrations (0.5, 1.0, or 2.0 μg/ml), cells were fixed and stained with DAPI before CLSM imaging. Representative images are shown from 3 independent experiments. (**B**) Average sizes of ataxin-1 NBs corresponding to the conditions as per (**A**) were measured using CellProfiler. Results represent the mean ± SEM (n > 70). Significance values calculated by ANOVA, **p < 0.01, ****p < 0.0001. (**C**–**E**) At 24 h post-transfection, cells were incubated in an imaging chamber equilibrated with 5% CO_2_ at 37 °C prior to FRAP using CLSM. (**C**) Representative images are shown from 3 independent experiments with FRAP assessments of exchange dynamics of GFP-ataxin-1[85Q] for different size NBs (denoted as I with diameter ≤0.75 μm, II with diameter 0.75–2 μm, and III diameter >2 μm). White rectangles indicate the ataxin-1 NBs analyzed; white open arrowhead indicates photobleached area. All scale bars = 10 μm. (**D**) Plot of the percentage recovery of fluorescence from experiments as shown in (C I-III) Each symbol represents fluorescence measured at the indicated time for the indicated ROI. (**E**) Recovery initial rates (average percentage recovery of fluorescence in the first 15 s) (Fn%/s) were calculated and shown by the pooled data. Each symbol represents a single data point obtained from one ROI across 3 independent experiments. Results represent mean ± SEM (n > 7 measured ROIs). Significance values were calculated by ANOVA, *p < 0.05.

PolyQ proteins can form larger protein structures that have been implicated as part of their toxicity mechanisms leading to neurodegeneration; this is clearly documented for the polyQ-huntingtin protein that forms heterogeneously-shaped nuclear aggregates^28^. In exploring the physical nature of the larger protein structures formed by polyQ-ataxin-1, we note that ataxin-1 NBs have been observed in SCA1 patients^29^ and that GFP-ataxin-1 forms distinctive NBs within the nucleoplasm of different cell lines^30,31^. Importantly, the remarkably spherical appearance of the ataxin-1[85Q] NBs (Fig. 1A, upper panel) raises the possibility that these NBs arise from phase separation of the ataxin-1[85Q] protein. Phase separation is a phenomenon that gives rise to membrane-less liquid-like compartments that are dependent on protein concentration^11,32^, are dynamic in composition^9^, and that display increased coordinated movement at the domain boundary due to the free energy cost to leave the compartment phase^33^. Thus, we exploited live cell imaging to explore these properties of the ataxin-1 NBs.

First, we expressed GFP-ataxin-1[85Q] in Neuro-2a cells at different levels by varying the concentrations of the transfected expression plasmid (0.5, 1.0 and 2.0 μg/ml plasmid), noting an increase in ataxin-1 NB size with increased transfected plasmid (Fig. 1A,B). To examine exchange dynamics for these ataxin-1 GFP-ataxin-1[85Q] NBs across these transfection conditions (Fig. 1C, rectangles, where I corresponds to NBs with a diameter ≤0.75 μm, II with a 0.75–2 μm diameter, III with a >2 μm diameter), we monitored fluorescence recovery after photobleaching (FRAP) (Fig. 1D). A rapid recovery of fluorescence for all NBs indicated a high exchange rate of GFP-ataxin-1[85Q] (Fig. 1C–E). Notably, the smaller NBs (group I) showed the fastest initial recovery rates (Fig. 1E) achieving near complete recovery within 20 s (Fig. 1D). Fluorescence recovery was demonstrated from either within the NB (i.e. non-bleached NB area) or the nucleoplasm surrounding the NB (Supp Fig. 1). These observations support our hypothesis that ataxin-1 NBs have liquid-like properties. Furthermore, during our FRAP analyses we frequently observed fusion events in which the movement of GFP-ataxin-1[85Q] NBs through the nucleoplasm brought two spherical NBs into close proximity, followed by their rapid fusion and re-shaping into a single, larger spherical NB (Supp. Fig. 2 and Supp. Movie 1). This observation suggests an ataxin-1 NB domain boundary that spatially confines diffusion, and upon fusion with another NB, undergoes a change in interfacial tension that permits mixing before re-establishment of a larger phase-separated compartment.

**Figure 2:**
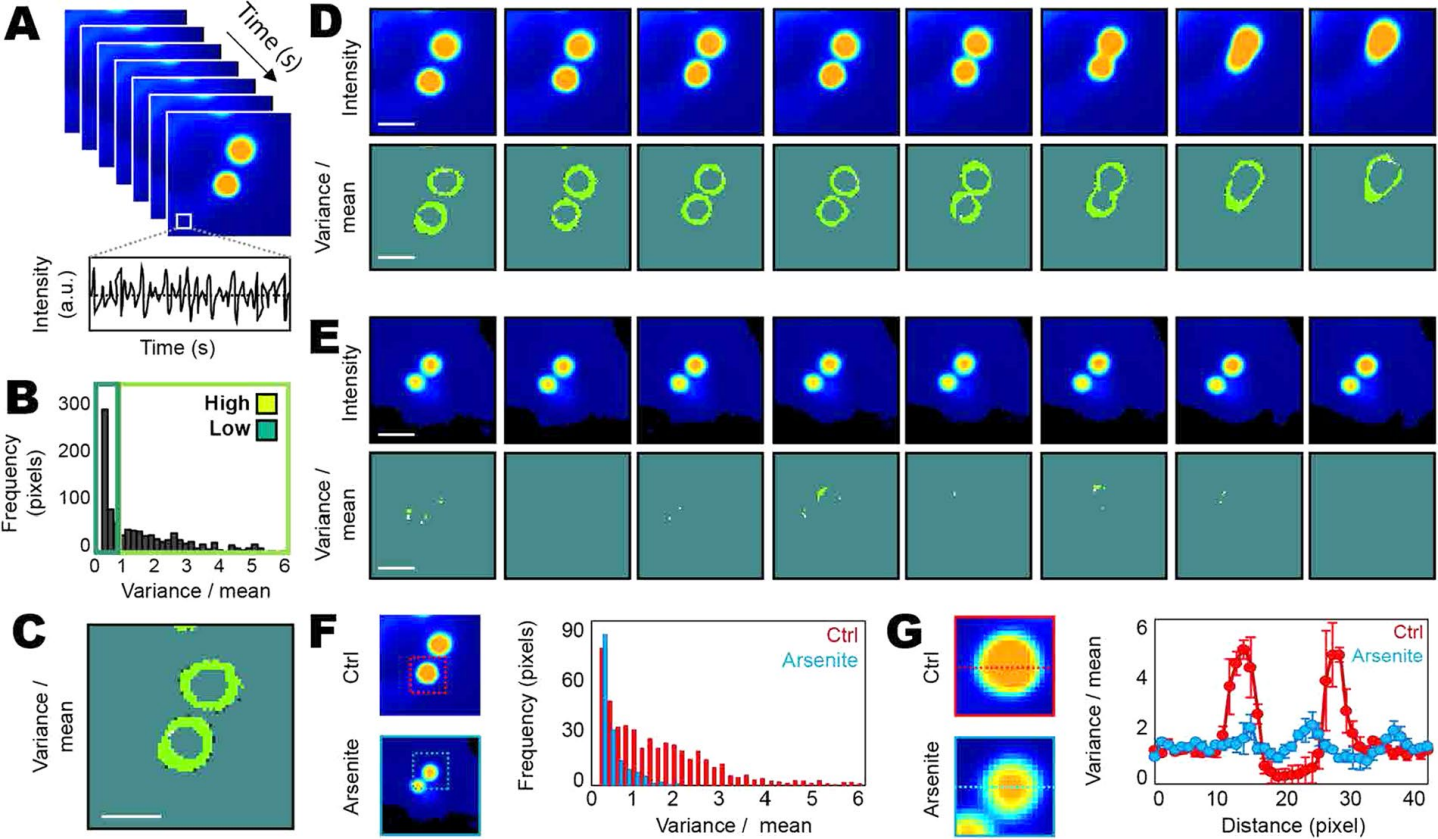
Fluorescence Fluctuation Spectroscopy (FFS) analysis of ataxin-1 NB fusion reveals liquid droplet-like behavior. Neuro-2a cells were transfected to express GFP-ataxin-1[85Q] for live cell imaging. (**A**) A time series acquisition (1000 frames) of ataxin-1 NBs enables the fluctuation in GFP-ataxin-1[85Q] fluorescence intensity to be acquired in each pixel of an image. (**B**,**C**) Estimate of coordinated GFP-ataxin-1[85Q] mobility in each pixel location. (**B**) Calculation of the ratio of the first and second moment (variance/mean) of the fluorescence fluctuation recorded in each pixel, directly corresponding to the image presented in (**C**). (**C**) The spatial map of a low versus high ratio of the variance to the mean demonstrates coordinated GFP-ataxin-1[85Q] mobility is at the NB boundary. (**D**) Time series acquisition (1000 frames) of two ataxin-1 NBs under control (Ctrl) conditions undergoing fusion, segmented into 10 s intervals (80 frames): the top row presents the intensity maps of GFP-ataxin-1[85Q] localization during each time segment and the bottom row presents the maps of GFP-ataxin-1[85Q] coordinated movement during each time segment. (**E**) Time series acquisition (1000 frames) of two ataxin-1 NBs not undergoing fusion in arsenite-treated cells, segmented into 10 s intervals (80 frames): the top row presents intensity maps of GFP-ataxin-1[85Q] localization during each time segment and the bottom row presents maps of GFP-ataxin-1[85Q] coordinated movement during each time segment. (**F**,**G**) A comparison of the degree of coordinated GFP-ataxin-1[85Q] movement (variance/mean) within (**F**) and across the axis (**G**) of GFP-ataxin-1[85Q] NBs undergoing fusion in Ctrl cells versus NBs not undergoing fusion in arsenite-treated cells. All scale bars in (**C**–**E**) = 2 μm. Images presented in (**D**–**G**) are representative of n = 3 cells under each condition, with average data presented in histograms in (**F**) and (**G**). Results in (**G**) represent mean ± SEM (n = 3 cells).

To investigate the biophysical properties of the ataxin-1 NB domain boundary and whether it imparts coordinated movement that is disrupted upon fusion with another ataxin-1 NB, we employed fluorescence fluctuation spectroscopy (FFS). Traditionally developed for the detection of protein oligomerisation^34^, a moment-based analysis of the fluctuation in fluorescence intensity recorded in each pixel of a confocal image can detect the localisation of coordinated protein movement in the context of phase separation^33^. Thus, from rapid acquisition of a time series of GFP-ataxin-1[85Q] fluorescence intensity images during a NB fusion event (1000 frames, 120 ms/frame), we calculated the ratio of the variance (2^nd^ moment) to the mean (1^st^ moment) of GFP-ataxin-1[85Q] fluorescence fluctuations recorded in each pixel of 80 frame segments (~10 s) (Fig. 2A,B). This analysis enables us to derive a spatial map of where GFP-ataxin-1[85Q] undergoes coordinated movement within a NB (i.e. high variance to mean ratio, Fig. 2C), and to trace this feature as a function of time during NB fusion (Fig. 2D).

As seen in a comparison of the fluorescent intensity images of GFP-ataxin-1[85Q] localization (top row, Fig. 2D) with the maps of coordinated GFP-ataxin-1[85Q] mobility (bottom row, Fig. 2D), we observed a high variance to mean ratio only at the NB boundaries. Then, as the two NBs become physically closer and start to fuse, there is a loss of coordinated GFP-ataxin-1[85Q] movement at the point of contact, which results in the high variance to mean ratio localization surrounding a single, larger spherical NB. Interestingly, upon exposure of the cells to an environmental pro-oxidant stress agent such as arsenite^35,36^, that is well-established for driving formation of the biomolecular condensates known as stress granules^37–39^, we observe a significant reduction in coordinated GFP-ataxin-1[85Q] mobility at the edge of the NB boundary (Fig. 2E). From quantitation of the frequency at which pixels are detected with a high variance to mean ratio in untreated control versus arsenite-treated cells (Fig. 2F) and then plotting this parameter as a function of position along a NB axis (Fig. 2G), we demonstrate that arsenite treatment results in a five-fold reduction in protein mobility at the NB boundary. Thus, in agreement with the protein concentration (Fig. 1A,B) and FRAP (Fig. 1C,D) analyses, fluorescence fluctuation spectroscopy (FFS) demonstrates a critical phase-separation hallmark for ataxin-1 NBs.

In addition, arsenite treatment not only decreased ataxin-1 NB dynamic exchange, but also increased ataxin-1[85Q] NB size (Supp. Fig. 3). Specifically, many larger ataxin-1 NBs were visible (Supp. Fig. 3A) and quantitative analyses demonstrated a statistically significant increase in average NB size following arsenite treatment (Supp. Fig. 3B). As the total size of the population of ataxin-1[85Q] NBs per nucleus did not also significantly increase upon arsenite exposure (Supp. Fig. 3C), these results are consistent with increased fusion of the ataxin-1 NBs in the presence of arsenite. To explore these relationships further, we also examined the behavior of an ataxin-1[85Q] mutant with a deletion in its self-association domain (SAD)^40^ (amino acids 495–605, herein termed ataxin-1[85Q]∆SAD) (Supp. Fig. 4). In transgenic mice models, polyQ-ataxin-1[77Q]∆SAD was reported as toxic (also tested in Neuro-2a cells as shown in Supp. Fig. 4C), but without visible NBs^41^. In our cultured Neuro-2a cell system, we observed that ataxin-1[85Q]∆SAD retained the ability to NBs potentially due to the presence of other motifs (such as CT2AXH) outside of the SAD^42^, but these differed from those formed by wild-type ataxin-1[85Q] being significantly smaller in size and not being further impacted by arsenite treatment (Supp. Fig. 4). Thus, the formation of ataxin-1[85Q] NBs can be observed in the absence of the reported self-association domain, and when integrated with the previous *in vivo* studies^41^, these results suggest that these smaller sized NBs may retain the potential for neurotoxicity.

In addition to evaluating the NBs formed by ataxin-1[85Q], we examined the properties of NBs formed by the wild-type ataxin-1[30Q] protein. Expression of GFP-ataxin-1[30Q] protein in Neuro-2a cells showed the formation of spherical NBs indistinguishable morphologically from those observed for GFP-ataxin-1[85Q] (Supp. Fig. 5A), and consistent with previous studies^43,44^, thus defining higher ataxin-1 protein concentration as a major contributor to NB formation. Furthermore, ataxin-1[30Q] NBs showed high dynamics (Supp Fig. 5A–C) and fusion (Supp Fig. 5D) revealed by FRAP and live cell imaging, respectively. Taken together, these results demonstrate ataxin-1 protein phase separation as liquid droplets with high dynamics and fusion.

### RNA/transcription is required for polyQ-ataxin-1 phase separation

**Figure 3:**
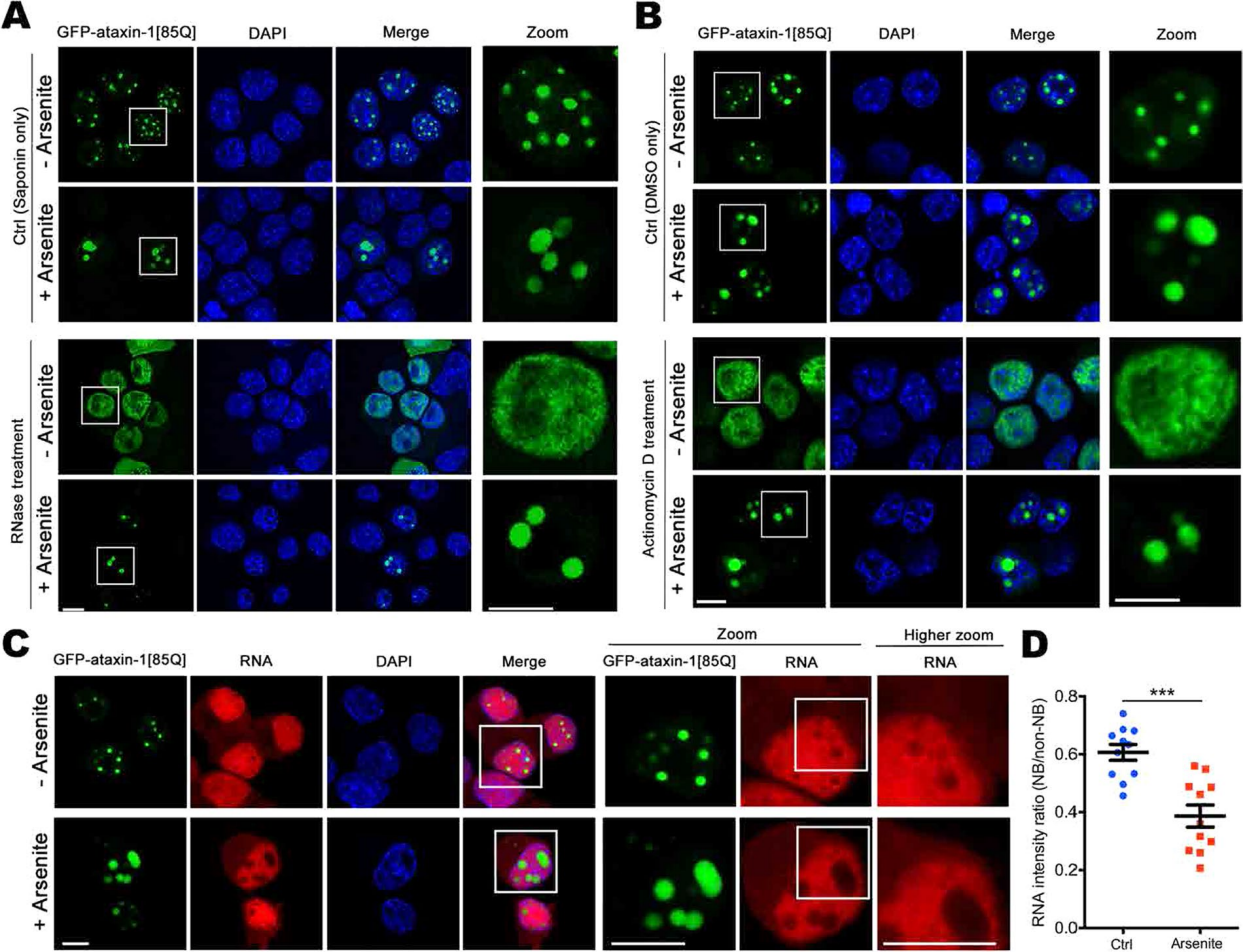
Ataxin-1 NBs are sensitive to RNase/transcription inhibition in control but not in arsenite stress conditions. Neuro-2a cells were transfected to express GFP-ataxin-1[85Q]. At 24 h post-transfection, cells were treated with arsenite as indicated (**A**–**C**), followed by treatment with (**A**) Saponin only (0.005%, 15 s) for membrane permeabilization, or Saponin + RNase A (100 μg/ml, 1 h, on ice) for RNA degradation, or (**B**) DMSO only, or Actinomycin D in DMSO (2 μg/ml, 3 h 37 °C). Cells were then fixed and stained with DAPI before CLSM imaging. Zoom images (right panels) correspond to the boxed regions. Representative images are shown from 3 independent experiments. (**C**) RNA synthesis was detected using imaging including the Click-iT RNA Alexa Fluor 594. 5-ethynyl uridine was added for 24 h before arsenite treatment, then incorporated 5-ethynyl uridine was detected (red). Zoom images (right panels) correspond to the boxed regions. (**D**) Quantitative analysis of RNA staining intensity ratio (NB area/non-NB area in the nucleoplasm). Each symbol represents a single data point. Results represent the mean ± SEM (n > 10). Significance values were calculated by unpaired two-tailed student’s t-test, ***p < 0.001. All scale bars = 10 μm.

We next explored the dependence of the formation of ataxin-1 NBs on the presence of RNA, building on previous studies demonstrating an interaction of ataxin-1 with RNA *in vitro*^45^ and also that RNA can contribute to protein phase separation^32,46^. We addressed this requirement for RNA/transcription in two independent protocols, and we further included an oxidant stress treatment by incubation of the cells in the presence of arsenite^35,36^. First, we assessed the impact of RNase-mediated degradation on ataxin-1 NBs. Pretreatment with the mild detergent saponin to permeabilise cell and nuclear membranes allowed RNase A access to hydrolyse RNA. Importantly, saponin treatment alone did not impact the ataxin-1 NBs, and so GFP-ataxin-1[85Q] was visualized within distinct NBs (Fig. 3A, upper two rows). In contrast, saponin followed by RNase A treatment resulted in a diffuse nuclear distribution of GFP-ataxin-1[85Q] for control cells; however, for cells previously treated with arsenite, we noted that the ataxin-1 NBs remained following RNase A treatment (Fig. 3A). We next considered the impact of the transcriptional inhibitor actinomycin D^47^. In control cells treated with actinomycin D for 3 h, we again noted a diffuse distribution of GFP-ataxin-1[85Q] in the nucleus; but for cells pre-treated with arsenite for 1 h, followed by washout and actinomycin D treatment for 3 h, we continued to observe distinct ataxin-1 NBs (Fig. 3B). We further examined ataxin-1 NBs for the presence of newly-synthesized RNA^48^. We noted intense staining for RNA throughout the nucleus; this included regions occupied by the GFP-ataxin-1[85Q] nuclear bodies, albeit at levels lower than in the surrounding nucleoplasm; under arsenite-treated conditions, we continued to detect RNA throughout the nucleus, without distinctive accumulation within ataxin-1 NBs (Fig. 3C). Indeed, quantitative analysis showed that arsenite treatment resulted in lower RNA levels in areas corresponding to the ataxin-1 NBs (Fig. 3D). These observations support a model in which low RNA levels are critical in initiating liquid droplet formation whereas higher RNA levels trigger droplet disassembly^49,50^. Thus, ataxin-1 NBs are less reliant on RNA levels when in the presence of arsenite stress.

### Different forms of stresses trigger phase transition of liquid ataxin-1 NBs to a reversible or an irreversible hydrogel

**Figure 4:**
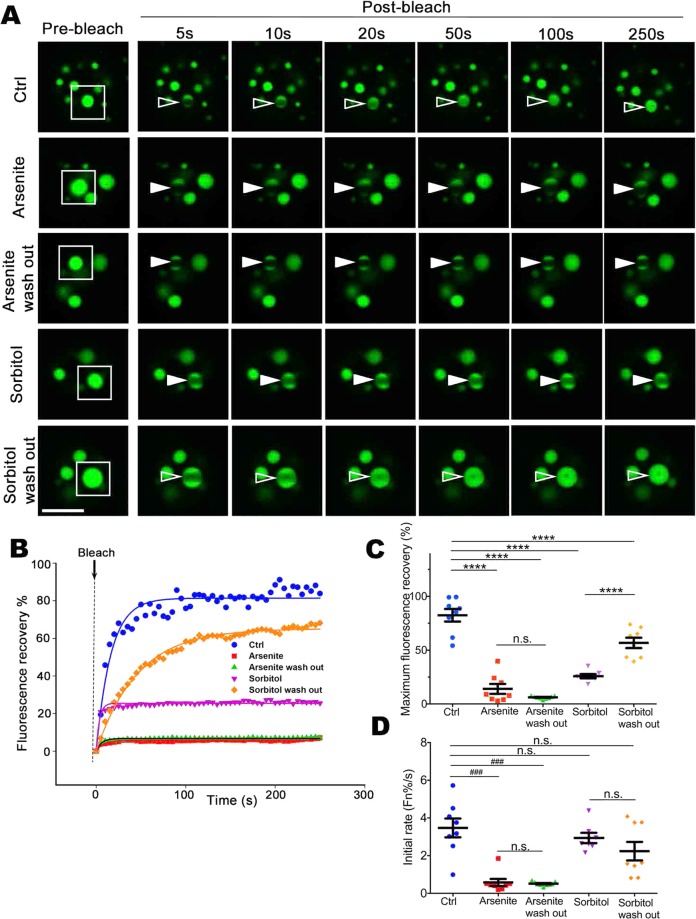
Different stresses can inhibit ataxin-1 NB exchange dynamics either irreversibly or reversibly. Neuro-2a cells were transfected to express GFP-ataxin-1[85Q]. At 24 h post-transfection, cells were left untreated (Ctrl) or treated with arsenite (0.3 mM, 1 h) (Arsenite) or sorbitol (0.5 M, 1 h) (Sorbitol). Following wash out (three washes with PBS), cells were left to recover from the stress treatment for 3 h (Arsenite wash out or Sorbitol wash out). (**A**) Representative images are shown from 3 independent experiments for quantitative assessments of GFP-ataxin-1[85Q] exchange dynamics. White rectangle indicates ataxin-1 NB imaged analyzed. A small ROI (indicated by white open arrowhead for dynamic NB or white solid arrowhead for non-dynamic NB) was photobleached and the fluorescence recovery was subsequently monitored as post-bleach at 5 s intervals for 250 s. (**B**) Plot of the percentage recovery of fluorescence from experiments as shown in (**A**). Each symbol represents fluorescence measured at the indicated time. (**C**,**D**) Results for pooled data for FRAP experiments. The recovery of fluorescence for GFP-ataxin-1[85Q] over time was analyzed. Each symbol represents a single data point. Results represent the mean ± SEM for (**C**) fluorescence maximum recovery percentage in the bleached area (where 100% represents full recovery) and (**D**) the initial rate of fluorescence recovery percentage in the bleached area for first three time points (0–15 s of post-bleach). Significance values were calculated by (**C**) ANOVA or (**D**) Mann-Whitney and Kruskal-Wallis non-parametric test, n > 7, ****p < 0.0001, ^###^p < 0.001, n.s. = not significant. All scale bars = 10 μm.

The liquid droplets formed by proteins with low complexity region (LCR) or prion-like domain (PrLD) motifs can show a time-dependent transition through an aging/maturation process^20,51^. Furthermore, exposure of cells to a range of environmental stress agents, such as arsenite, can enhance stress granule formation^37–39^. Therefore, we next assessed the impact of arsenite on the maturation/phase transition of ataxin-1 NBs. Our FRAP analyses revealed a significant slowing of GFP-ataxin-1[85Q] maximum recovery and initial rate of recovery, an impact not reversed upon arsenite washout (Fig. 4).

**Figure 5:**
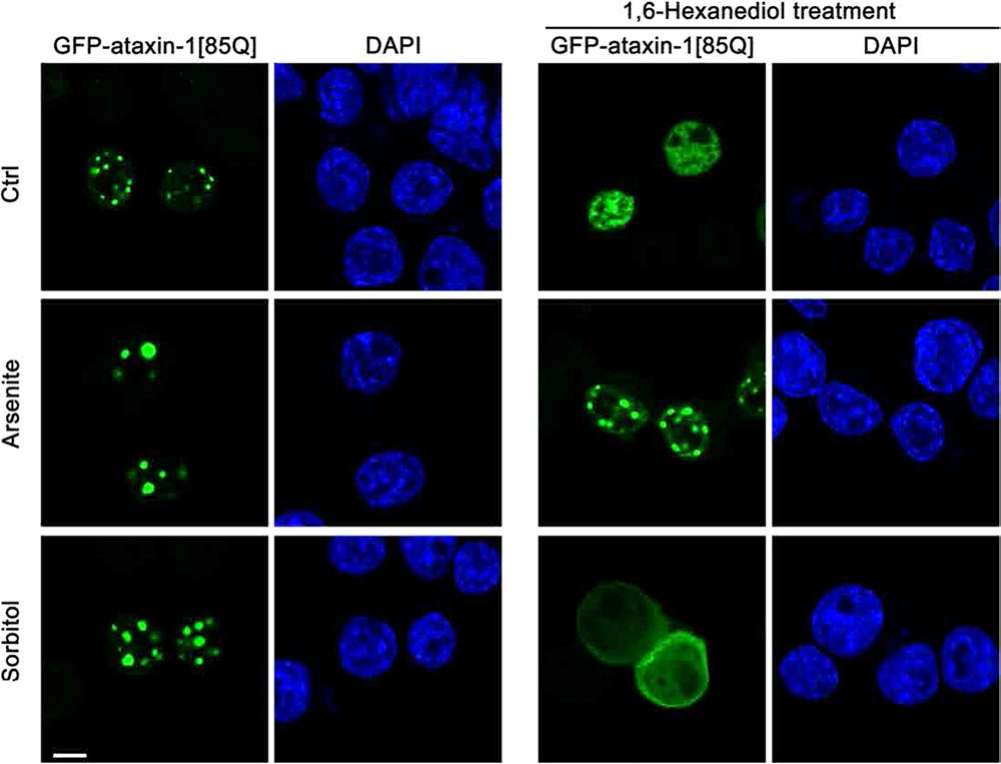
Liquid-like or solid-like properties of ataxin-1 NBs under control or stress conditions. Neuro-2a cells were transfected to express GFP-ataxin-1[85Q]. At 24 h post-transfection, cells were left untreated (Ctrl) or treated with arsenite (0.3 mM, 1 h) (Arsenite) or sorbitol (0.5 M, 1 h) (Sorbitol). Cells were further treated with 1,6-hexanediol (5%, 5 min) to discriminate between liquid-like assemblies and solid-like assemblies. Cells were then fixed and stained with DAPI before CLSM imaging. Each symbol represents a single data point. Representative images are shown from 3 independent experiments. All scale bars = 10 μm.

We extended our interrogations to include a second environmental insult in the form of hyperosmotic stress induced by addition to the cell culture medium of the non-metabolisable sugar, sorbitol; this change in osmolarity can also promote the formation of cytoplasmic stress granules^52,53^. We again observed significant slowing of GFP-ataxin-1[85Q] maximum recovery and initial rate of recovery, but these changes could be reversed upon the return of the cells to normal osmotic conditions (Fig. 4). Thus, the impact of stress on the transition to form non-dynamic ataxin-1 NBs is dependent on the nature of the initiating stress. This altered physical state of the ataxin-1 NBs does not appear to be caused by altered levels of ataxin-1 expression under these stress conditions, with no significant differences in total fluorescence intensity observed across the control, arsenite, or sorbitol treatments (Supp. Fig. 6). To further test the state of the ataxin-1 NBs under different conditions, 1,6-hexanediol, a chemical probe to differentiate liquid-like assemblies from solid-like assemblies^8,54^, was added to the cells expressing GFP-ataxin-1[85Q] under control or stress conditions (arsenite or sorbitol). Whilst ataxin-1 NBs exhibit a liquid-like state sensitive to 1,6-hexanediol, NBs pre-treated with stress (arsenite but not sorbitol) exhibit a solid-like state as they are 1,6-hexanediol resistant (Fig. 5). Thus, ataxin-1 protein exchange and the properties of ataxin-1 NBs are rapidly responsive to environmental changes.

### Maintenance of the ataxin-1 NB liquid phase requires ATP

**Figure 6:**
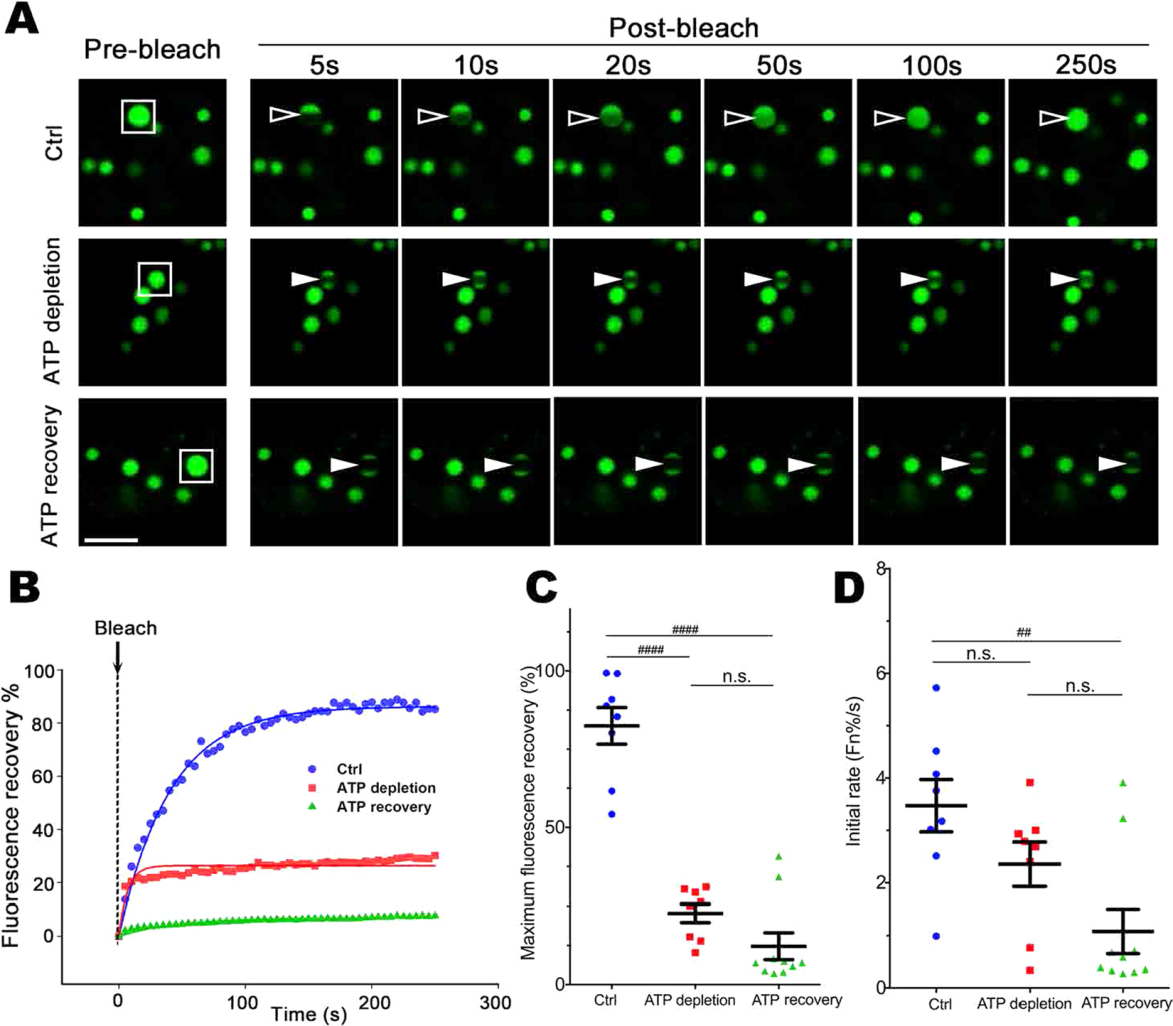
Dynamic ataxin-1 NBs transit to irreversible non-dynamic hydrogel following intracellular ATP depletion. Neuro-2a cells were transfected to express GFP-ataxin-1[85Q]. At 24 h post-transfection, cells were left untreated (Ctrl) or treated with 2DG + CCCP (ATP depletion). Inhibitors were washed out (3 washes with PBS) and cells were left to recover for 3 h (ATP recovery). (**A**) Representative images are shown from 3 independent experiments for quantitative assessments of GFP-ataxin-1[85Q] exchange dynamics. White rectangle indicates ataxin-1 NB analyzed. A small ROI (indicated by white open arrowhead for dynamic NB or white solid arrowhead for non-dynamic NB) was photobleached and the fluorescence recovery was subsequently monitored as post-bleach at 5 s intervals for 250 s. (**B**) Plot of the percentage recovery of fluorescence from experiments as shown in (**A**). Each symbol represents fluorescence measured at the indicated time. (**C**,**D**) Results for pooled data for FRAP experiments. The recovery of fluorescence for GFP-ataxin-1[85Q] over time was analyzed. Each symbol represents a single data point. Results represent the mean ± SEM for (**C**) fluorescence maximum recovery percentage in the bleached area (where 100% represents full recovery) and (**D**) the initial rate of fluorescence recovery percentage in the bleached area for first three time points (0–15 s of post-bleach). Significance values were calculated by Mann-Whitney and Kruskal-Wallis non-parametric test, n > 7, ^##^p < 0.01, ^####^p < 0.0001, n.s. = not significant. All scale bars = 10 μm.

The maintenance of liquid droplet high dynamics, for membrane-less organelles such as nucleoli, is ATP-dependent^7^. Therefore, we next addressed the ATP requirement in the dynamic exchange of GFP-ataxin-1[85Q] in ataxin-1 NBs. We lowered ATP levels with 2-Deoxy-D-glucose (2DG) and Carbonyl cyanide m-chlorophenyl hydrazone (CCCP)^55,56^ and confirmed ATP recovery upon inhibitor washout (Supp. Fig. 7). Strikingly, maximum and initial rates of fluorescence recovery were significantly and irreversibly inhibited by ATP depletion (Fig. 6A–D), indicating the irreversibility of the phase transition process following ATP depletion.

### Down regulation of DEAD/H-box RNA helicases decreases ataxin-1 NB dynamic exchange

Whilst ATP can directly influence the phase transition process^57,58^, intracellular ATP levels are also critical for the actions of multiple ATP-dependent enzymes and chaperones^59,60^. We previously identified 13 RNA helicases in the ataxin-1 interactome (see ProteomeXchange, PXD010352)^61^. RNA helicases have ubiquitous roles in regulating RNA structures as well as RNA-RNA and RNA-protein interactions in multiple different aspects of RNA metabolism, including transcription, mRNA splicing, RNA export, translation and RNA degradation^62,63^. The two largest groups within the RNA helicase family are the DEAD-box (DDX) and DEAH-box (DHX) helicases^63^. That these RNA helicases may contribute to protein phase transition of membrane-less NBs is seen with siRNA-driven decrease in RNA helicase CGH-1/DDX6 levels resulting in non-dynamic ribonucleoprotein (RNP) granules^64^.

**Figure 7:**
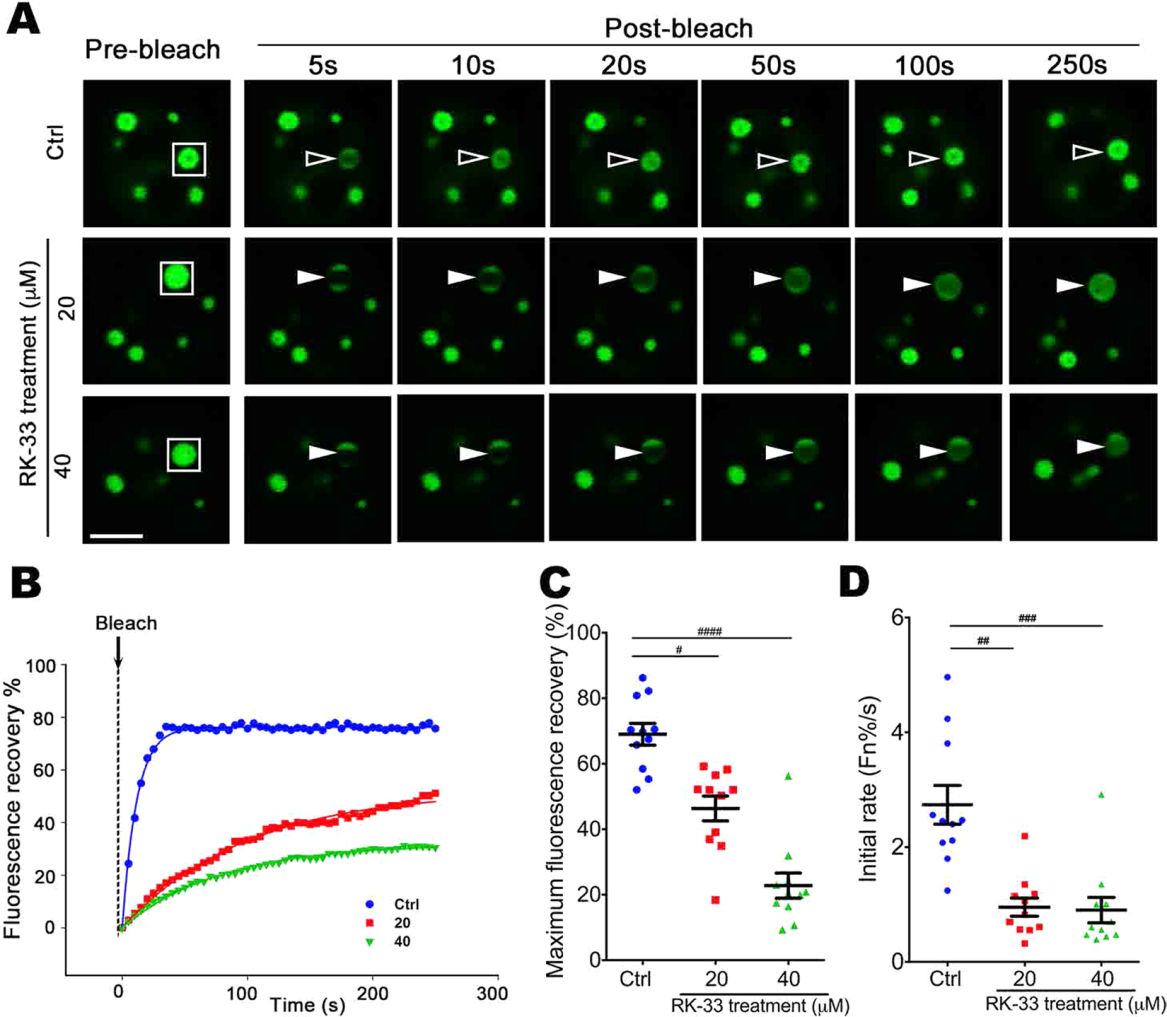
RNA helicase inhibitor RK-33 slows ataxin-1 NB exchange dynamics. Neuro-2a cells were transfected to express GFP-ataxin-1[85Q]. At 4 h post-transfection, cells were left untreated (Ctrl) or treated with RK-33 (20 μM or 40 μM) for 24 h. (**A**) Representative images are shown from 3 independent experiments for quantitative assessments of GFP-ataxin-1[85Q] exchange dynamics. White rectangle indicates ataxin-1 NB analyzed. A small ROI (indicated by white open arrowhead for dynamic NB or white solid arrowhead for non-dynamic NB) was photobleached and the fluorescence recovery was subsequently monitored as post-bleach at 5 s intervals for 250 s. (**B**) Plot of the percentage recovery of fluorescence from experiments as shown in (**A**). Each symbol represents fluorescence measured at the indicated time. (**C**,**D**) Results for pooled data for FRAP experiments. The recovery of fluorescence for GFP-ataxin-1[85Q] over time was analyzed. Each symbol represents a single data point. Results represent the mean ± SEM for (**C**) fluorescence maximum recovery percentage in the bleached area (where 100% represents full recovery) and (**D**) the initial rate of fluorescence recovery percentage in the bleached area for first three time points (0–15 s of post-bleach). Significance values were calculated by Mann-Whitney and Kruskal-Wallis non-parametric test, n > 10, ^#^p < 0.05, ^##^p < 0.01, ^###^p < 0.001, ^####^p < 0.0001. All scale bars = 10 μm.

**Figure 8:**
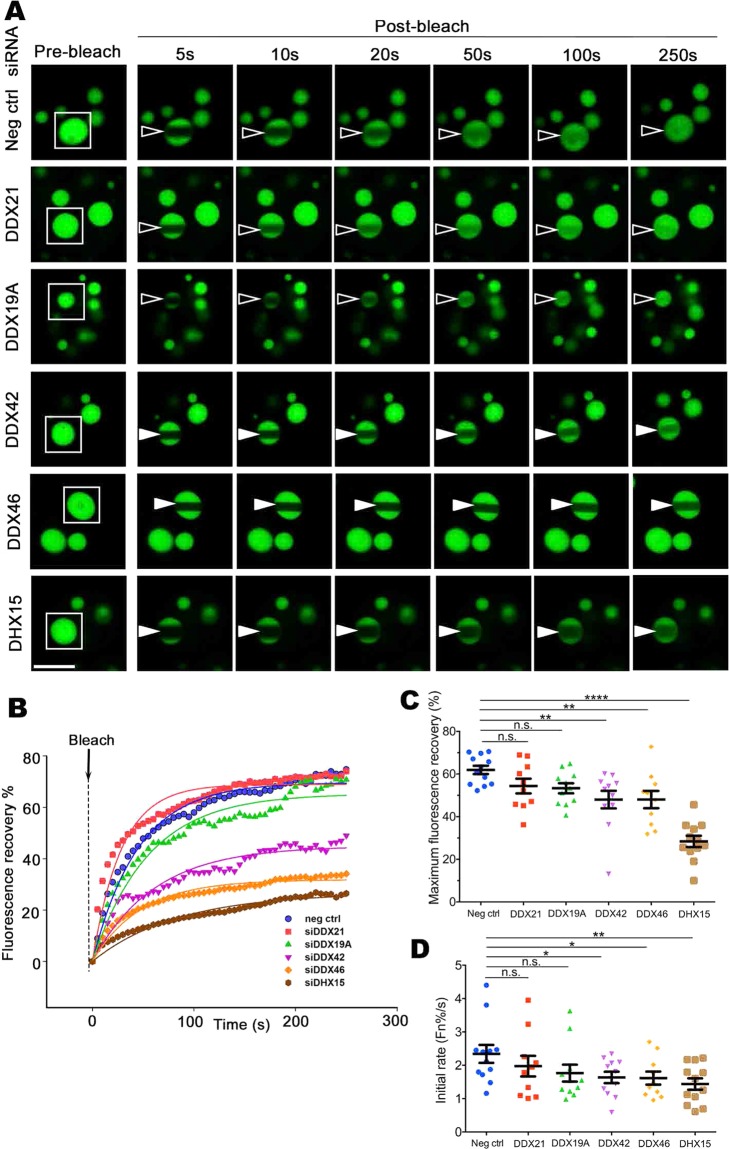
ATP-dependent RNA helicases DDX42, DDX46 and DHX15 are required for ataxin-1 NB rapid exchange dynamics. Neuro-2a cells were co-transfected with plasmid encoding GFP-ataxin-1[85Q] and siRNAs: negative siRNA ctrl, DDX21, DDX19A, DDX42, DDX46, or DHX15, as indicated. At 24 h post-transfection, cells were incubated in an imaging chamber equilibrated with 5% CO_2_ at 37 °C prior to FRAP using CLSM. (**A**) Representative images are shown from 4 independent experiments for quantitative assessments of GFP-ataxin-1[85Q] exchange dynamics under different siRNA conditions. White rectangle indicates ataxin-1 NB analyzed. A small ROI (indicated by white open arrowhead for dynamic NB or white solid arrowhead for non-dynamic NB) was photobleached and the fluorescence recovery was subsequently monitored as post-bleach at 5 s intervals for 250 s. (**B**) Plot of the percentage recovery of fluorescence from experiments as shown in (**A**). Each symbol represents fluorescence measured at the indicated time. (**C**,**D**) Results for pooled data for FRAP experiments. The recovery of fluorescence for GFP-ataxin-1[85Q] over time was analyzed. Each symbol represents a single data point. Results represent the mean ± SEM for (**C**) fluorescence maximum recovery percentage in the bleached area (where 100% represents full recovery) and (**D**) the initial rate of fluorescence recovery percentage in the bleached area for first three time points (0–15 s of post-bleach). Significance values were calculated by ANOVA, n > 10, *p < 0.05, **p < 0.01, **** p < 0.0001, n.s. = not significant. All scale bars = 6 μm.

We initially showed that RK-33, an inhibitor that binds the ATP-binding cleft of RNA helicase(s)^65,66^, could decrease the exchange dynamics of ataxin-1 NBs (Fig. 7). We next incubated cells in the presence of siRNAs targeting each of the 13 RNA helicases of the ataxin-1 interactome. Our initial FRAP assessments directed our focus to siRNA targeting of the helicases DDX21, DDX19A, DDX42, DDX46, and DHX15. We ensured that the levels of each, as assessed by Western blot, were decreased by siRNA treatment (Supp. Fig. 8). Using FRAP protocols, we assessed how these helicase-targeted siRNA treatments, alongside control siRNA treatment, altered the dynamics of recovery. Our quantitative analysis of the pooled data confirmed that DDX42, DDX46, or DDX15 siRNA significantly decreased the dynamic exchange of the ataxin-1 NBs (Fig. 8) by decreasing both the maximum fluorescence recovery (Fig. 8C) and the initial recovery rate (Fig. 8D). When we assessed the intracellular localization of each helicase, we further noted that DDX42, DDX46, and DHX15 were more nuclear than cytoplasmic (Supp. Fig. 9A,B). Taken together, these results reinforce the observation that DDX42, DDX46 and DHX15 are predominantly nuclear RNA helicases that contribute to the regulation of the dynamic exchange of ataxin-1 NBs.

The processes of protein phase transition have been previously discussed as part of a time-dependent or “aging” process that can be linked to neurodegeneration. For example, in ALS, protein condensates formed by FUS or hnRNPA1 can transit to a more solid hydrogel or aggregate phase^8,9,19^. Initially this process can be reversed by ATP-dependent chaperones or disaggregases^67^, but the increasing spatial order of these protein assemblies then promotes a transition to a solid-like fibrous aggregate, that may be toxic for neurons and so be a driver of ALS neurodegeneration^14^. We therefore tested the impact of down regulation of RNA helicases on cell viability as an approach to defining whether RNA helicase sequestration/disruption might underpin ataxin-1[85Q]-driven cell toxicity. Treatment of the cells with siRNAs directed to the helicases DDX42, DDX46, or DHX15 that decrease ataxin-1 NBs dynamics (Fig. 8) did not significantly increase cell death percentage in GFP-expressing cells, and indeed there was a small but significant increase in viability noted for the DDX42 siRNA-treated GFP-expressing cells (Supp Fig. 10). This demonstrates that a disruption of RNA helicases, and in particular the single disruption of DDX42, DDX46 or DHX15, is not a toxic event *per se*, but it also further suggests a decreased dynamics of ataxin-1[85Q] NBs that is observed upon the down regulation of specific RNA helicases is not caused by reduced cell viability.

### Arsenite increases ataxin-1[85Q]S776A toxicity and decreases ataxin-1[85Q]S776A NB dynamic exchange

**Figure 9:**
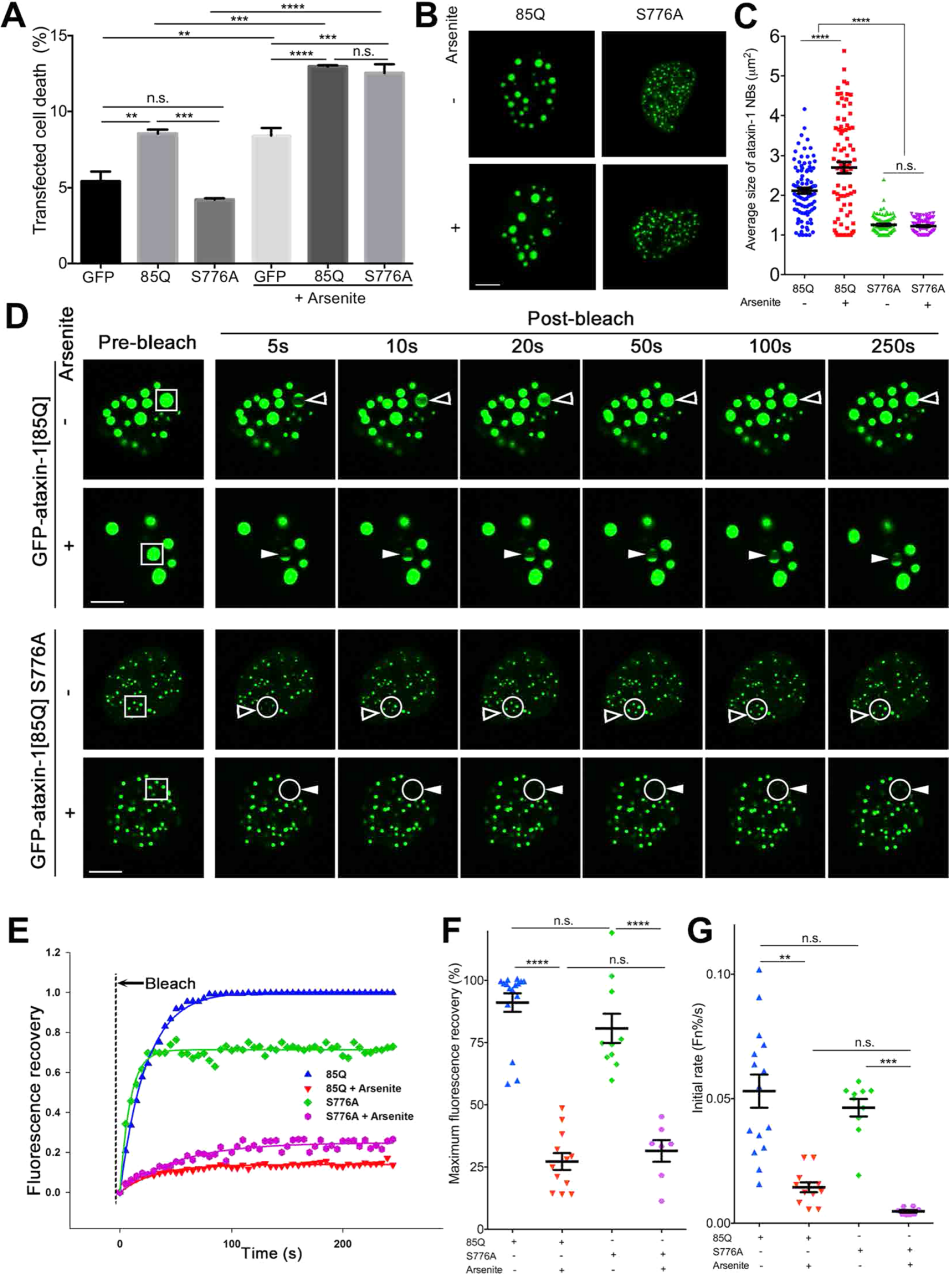
Non-toxic ataxin-1 mutant S776A shows increased toxicity and decreased NB exchange dynamics similar to ataxin-1[85Q] level following arsenite stress. Neuro-2a cells were transfected with GFP, GFP-ataxin-1[85Q], or GFP-ataxin-1[85Q]S776A as indicated. At 24 h post-transfection, cells were left untreated or treated with arsenite (0.3 mM, 1 h). (**A**) Cell death percentage for the transfected cell population was assessed by staining with the SYTOX Red dead cell stain followed by flow cytometry analysis. (**B**,**C**) Cells were fixed and stained with DAPI before CLSM imaging. Representative images are shown from 3 independent experiments. (**C**) Average sizes of ataxin-1 NBs corresponding to the conditions as per (**B**) were measured using CellProfiler. Results represent the mean ± SEM (Significance values calculated by ANOVA, n > 70, ****p < 0.0001, n.s. = not significant). (**D**–**G**). cClls were incubated in an imaging chamber equilibrated with 5% CO_2_ at 37 °C prior to FRAP using CLSM. (**D**) Representative images are shown from 3 independent experiments for quantitative assessments of GFP-ataxin-1[85Q] or S776A exchange dynamics. A small ROI (indicated by white open arrowhead for dynamic NB or white solid arrowhead for non-dynamic NB; S776A ROI with multiple small NBs was indicated also by the white circle) was photobleached and the fluorescence recovery was subsequently monitored as post-bleach at 5 s intervals for 250 s. Scale bars = 10 μm. (**E**) Plot of the percentage recovery of fluorescence from experiments as shown in (**D**). Each symbol represents fluorescence measured at the indicated time. (**D**–**E**) Pooled data from FRAP experiments measuring recovery of fluorescence for GFP-ataxin-1[85Q] or GFP-ataxin-1[85Q] S776A over time. Each symbol represents a single data point. The results are also represented as the mean ± SEM for (**F**) fluorescence maximum recovery percentage in the bleached area (where 100% represents full recovery) and (**F**) initial rates (average percentage recovery of fluorescence in the first 15 s) (Fn%/s). Significance were values calculated by ANOVA, n > 7, ^***^p < 0.0001, ^****^p < 0.001, n.s. = not significant.

To further explore any correlation between NB size and dynamics and cell toxicity, an ataxin-1 S776A mutant (ataxin-1 serine 776 mutated to the non-phosphorylatable alanine)^68,69^ was examined (Fig. 9). Indeed, ataxin-1[85Q]S776A remained non-toxic under control conditions as reported^68,69^ with levels of cell death being not statistically different from those recorded for GFP-only transfected cells (Fig. 9A). However, upon arsenite treatment, we observed statistically significant increases in death of cells expressing GFP-only, GFP-ataxin-1[85Q] or GFP-ataxin-1[85Q]S776A such that toxicity for cells expressing either form of ataxin-1 was comparable (Fig. 9A). When we examined the morphology of ataxin-1 protein exchange for the NBs formed, we observed that ataxin-1[85Q]S776A formed smaller NBs that did not increase in size upon arsenite treatment (Fig. 9B). This suggests that size of the formed NB cannot itself be used as a predictor of toxicity, and thus we further considered the dynamics of these NBs, noting the similar level of high dynamics of ataxin-1 exchange for both the ataxin-1[85Q] and ataxin-1[85Q]S776A NBs, and with the dynamic exchange of both significantly slowing upon arsenite exposure (Fig. 9E–G). These results suggest that decreased NBs dynamics may be related to enhanced cytotoxicity, but this interpretation may be complicated by the broad spectrum of impacts of arsenite exposure^35–39^.

## Discussion

**Figure 10:**
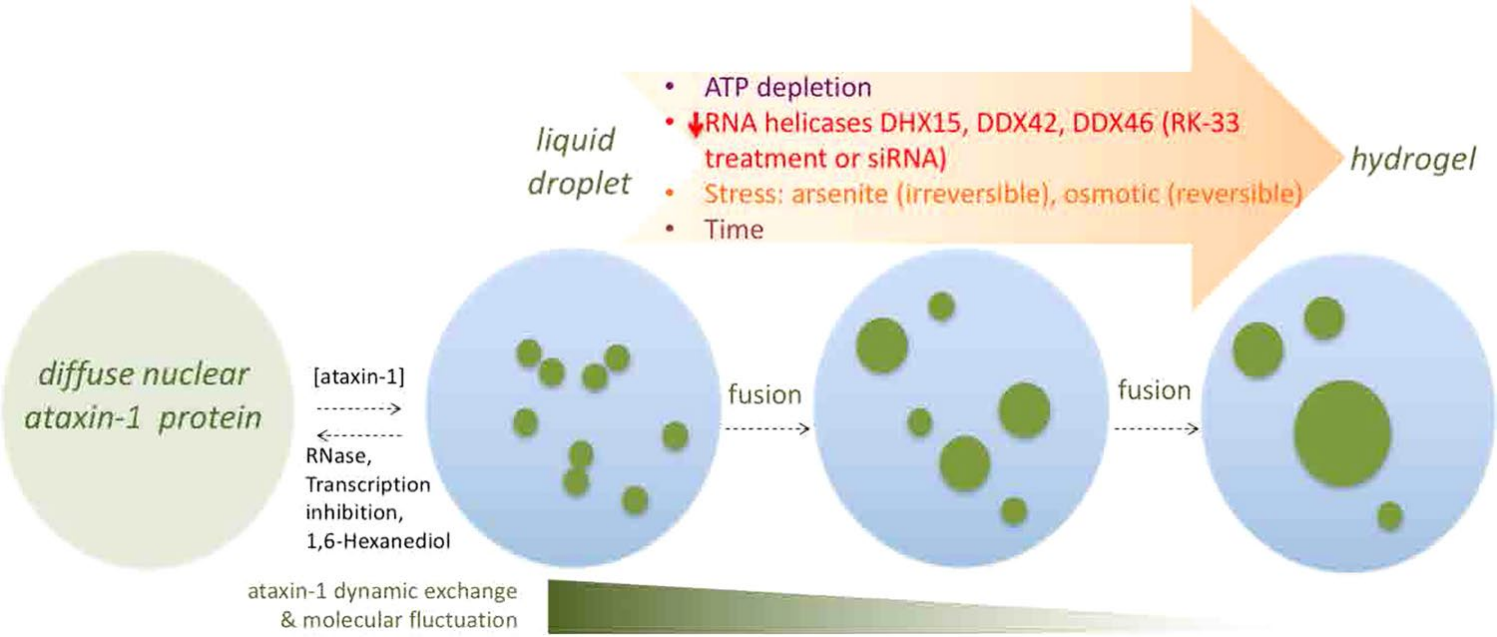
Model showing the multiple factors contributing to the tunable dynamics of ataxin-1 NBs. Ataxin-1 can transit through three stages: diffuse nuclear protein, liquid droplet and then hydrogel, with multiple factors controlling these transitions. Initially, perturbations in ataxin-1 protein level or RNA/transcription, or by 1,6-hexanediol, can influence the transition from diffuse protein to dynamic liquid droplets that are visualized as distinct spherical ataxin-1 NB; fusion can result in larger NBs, but additional factors of time, altered RNA helicases by inhibitor RK-33 or siRNA, ATP depletion, or environmental stresses impact these ataxin-1 NBs to establish a less exchange dynamic and molecular fluctuation hydrogel state.

Proteins with LCR or PrLD have attracted much recent attention due to their ability to phase separate to liquid droplets and transition to disease-associated non-dynamic aggregates^8,9,19^. Here we interrogate polyQ-ataxin-1 NBs in cells to show, for the first time, their multiple liquid-like properties and phase transition to structures with lower dynamic exchange. Based on our findings of NB fusion and the altered protein exchange dynamics we propose a model, as depicted in Fig. 10, in which the ataxin-1 protein undergoes phase separation and then transitions via the liquid droplet state to ultimately form a less dynamic state typical of a hydrogel. In the following paragraphs, we discuss key features of this model and its integration with the current concepts of the formation of biomolecular condensates and the reported physical features and toxicity of polyQ proteins.

Initially, the formation of NBs is favoured by an increased concentration of the ataxin-1 protein itself (Fig. 1). Whilst proteins at low concentrations show diffuse intracellular distributions, phase separation can be observed when a critical threshold concentration intrinsic to each protein is exceeded^8,11–13,19^. This concentration-dependent phase separation has been reported for multiple LCR/PrLD-containing proteins, including ALS disease-causing proteins FUS, hnRNPA1, TIA1, and polyQ protein WHI3^12^; because the polyQ tract is a predicted PrLD^21,22^, polyQ-ataxin-1 is expected to behave in this way in cells. Consistent with previous reports^70^, here we have shown that both mutant polyQ-ataxin-1 (with an expanded polyQ tract) and wild-type ataxin-1 protein (with a shorter polyQ tract) can form NBs (Fig. 1 and Supp. Fig. 3). Importantly, although the toxic form of ataxin-1 was first identified with an expanded polyQ tract^23,71^, increased expression of even the wild-type ataxin-1 causes SCA1 phenotypic changes in flies and mice models^43,44^. Thus, increased ataxin-1 protein levels are important in the toxicity mechanisms in SCA1 and a significant consequence of an expanded polyQ tract is increased stability that favours the accumulation of higher levels of ataxin-1^72^ and thus the formation of ataxin-1 NBs.

RNA also contributes to the processes of phase separation for ataxin-1 as demonstrated by our results showing the impact of RNase treatment or actinomycin D exposure to drive diffuse distribution of the ataxin-1 protein mainly in the nucleus (Fig. 3). Whilst RNA alone can undergo phase separation into liquid droplets^10^, RNA can also change the material properties of protein assemblies to influence phase separation and transition^12,32,73^. Ataxin-1’s ability to interact with RNA *in vitro* has been reported as showing strong binding to poly(rG) but weaker binding to poly(rU), poly(rA), and poly (rC)^45^. Our bioinformatics assessment of the ataxin-1 interactome^61^ highlights a link of ataxin-1 to the processes of RNA metabolism and specifically identified multiple RNA helicases. Our identification of the RNA helicases DDX42, DDX46, and DHX15 which function in pre-mRNA processing^74–76^ as regulators of ataxin-1 NBs dynamics (Figs. 7 and 8) reinforces a functional association of ataxin-1 with RNA metabolism^68,77^.

We also characterized the liquid droplet state of the ataxin-1 NBs (Figs. 1 and 2). Importantly, our FFS analyses revealed that the ataxin-1 NB boundary imparts coordinated movement and that upon fusion of two individual ataxin-1 NBs the interfacial tension established by this boundary is disrupted such that mixing between the two compartments is permitted and a single larger NB is formed. The application of FFS to quantify molecular mobility within intracellular compartments has contributed important new insights into accessibility of nuclear structures and the properties of biomolecular condensates, including the heterochromatin domain formed by phase separation^33,78,79^. By capitalizing on the capacity of FFS to detect the localisation of coordinated protein movement, we recover a map that reports the coordinated movement of ataxin-1 in and out of the NB, which can be tracked as a function of time and modified by arsenite stress (Fig. 2). Thus, FFS should be considered a critical inclusion in the approaches to study biomolecular condensates.

Importantly, our use of FRAP protocols revealed the tunable dynamics of ataxin-1 exchange for these NBs; exchange under control conditions was rapid, but multiple factors could slow this exchange. This reinforces the concepts of phase transition in which liquid-like condensates show rapid fusion and high dynamics, but then the processes of phase transition result in the formation of non-dynamic hydrogels, and ultimately solid-like fibrous aggregates, characterized by a higher spatial order of protein structures and decreased exchange dynamics with their environment^14^. For ataxin-1 NBs, we showed that this characteristic of the phase transition process could be promoted by the exposure of the cells to different forms of environmental stress (Figs. 4 and 5) or ATP depletion (Fig. 6). These changes echo the observations of previous studies that highlighted a maturation of dynamic ataxin-1 NBs over time to result in mixed populations with slow- or fast-exchanging components^70^, and support the notion that external factors such as temperature, pH, salinity, oxidative stress or osmolarity can promote phase separation and transition^12,80^. The application of arsenite or sorbitol stress is known to induce the formation of the biomolecular condensates known as stress granules^37–39,52,53^ by causing pro-oxidative or hyperosmotic stress responses, respectively, that ultimately stall protein translation^37,53^. Here we have noted a striking difference for ataxin-1 NBs: the transition to the non-dynamic hydrogel state is irreversible if initiated by arsenite, but reversible if initiated by osmotic stress. The exact mechanisms underlying these differences require further evaluation, but may reflect irreversible oxidative damage in the presence of arsenite, but reversible osmotic control via water efflux and influx^81^ when osmolarity is modulated by the presence of different concentrations of sorbitol.

Our study has provided the first comprehensive evaluation of the physical properties of these NBs (Figs. 1–6), evaluating a suite of manipulations that allow us to define a relationship between NB dynamic exchange and toxicity (Figs. 7, 8, Supp. Figs. 9 and 10). Thus, we propose that possible links between the regulation of ataxin-1 NB dynamics and the neurodegeneration observed in SCA1 patients should be an important area for future investigation. A dynamic exchange in the liquid droplet stage of the ataxin-1 NB formation would not restrict the transport of associated proteins into and/or exiting the NB^27^; thus ataxin-1 NBs are disassembled during mitosis and then ataxin-1 protein can be re-distributed into two cells^82^. With the phase transition to an irreversible hydrogel as that caused by ATP depletion or arsenite stress, it is inevitable that cellular function would be impacted if additional proteins essential for normal cellular functions are sequestered. Current SCA1 murine models (e.g. SCA1[82Q] transgenic mice^83–85^ or SCA1[154Q/2Q] knock-in mice^86–88^) rely on the assessment of downstream pathological impacts but, to date, have not included fluorescent protein tagging of the expressed polyQ-ataxin-1 protein. Thus, future studies in murine SCA1 models engineered to express fluorescent protein-tagged polyQ-ataxin-1 will enable the study of the dynamics of ataxin-1 NBs in Purkinje cells, for example by capitalizing on recent advances in lattice light sheet microscopy that offer advantages in spatial and temporal resolution as well as low phototoxicity^89,90^. The polyQ-ataxin-1 phase transition model that we propose (Fig. 10) will thus provide a framework for ongoing investigations to contribute new knowledge to a mechanistic understanding how polyQ-ataxin-1 can drive the pathological changes observed in the neurodegenerative disease SCA1.

## Materials and Methods

### Cell culture, transfection and treatments

Mouse neuroblastoma cells (Neuro-2a) were used in a majority of experiments and were cultured in growth medium: Opti-MEM (Gibco) supplemented with 2 mM L-glutamine (Gibco), 10% (v/v) fetal bovine serum (BOVOGEN) and 100 U/ml penicillin/streptomycin (Gibco). Where indicated in control experiments, Ad293 cells were cultured in Dulbecco’s Modified Eagle’s media (DMEM) supplemented with 10% [v/v] fetal bovine serum (FBS) (BOVOGEN) and 100 U/ml penicillin/streptomycin (Gibco). Cells were plated and cultured (16 h) for the following protocols: immunofluorescence and RNA staining (12-well plate, Corning), Western blot (6-well plate, Corning), live cell imaging (8-well μ-slides, Ibidi), and ATP measurements (96 well plate, Corning) then, transfected with the indicated GFP-ataxin-1[85Q] or GFP-ataxin-1[30Q] constructs using Lipofectamine 2000 reagent (Invitrogen) according to the manufacturer’s instructions (24 h). Unless indicated otherwise, 1 µg plasmid DNA/mL culture medium was used per transfection.

Cells transfected to express GFP-ataxin-1 proteins were subjected to different treatments as follows. For environmental stress, cells were exposed to either the pro-oxidant stress of arsenite (0.3 mM, 1 h, 37 °C) (Sigma), whereas for hyperosmotic stress cells were exposed to the non-metabolisable sugar D-sorbitol (0.5 M, 1 h, 37 °C) (Ajax Finechem). For RNase treatment, cells were treated with saponin (0.005%, 15 s, room temperature) (Sigma) prior to the addition of RNase A (100 μg/ml, 1 h, ice) (Invitrogen); thus, saponin-only treatment was employed in parallel control studies. For transcription inhibition, cells were exposed to actinomycin D (2 μg/ml, 3 h, 37 °C) (Sigma), with an equivalent exposure to only the vehicle DMSO (0.2%, 3 h, 37 °C) (Merck), as a negative control. For 1,6-hexanediol treatment, cells were incubated with 5% 1,6-hexanediol (5 min, RT) (Sigma) prior to cell fixation. For RK-33 treatment, cells were treated with 20 μM or 40 μM RK-33 (Adooq, A16194) (24 h, 37 °C) 4 h after transfection. For ATP depletion, cells were exposed to 2-deoxyglucose (2DG) (20 mM, 1 h, 37 °C) (Adrich Chem), + Carbonyl cyanide m-chlorophenyl hydrazine (CCCP) (100 μM, 1 h, 37 °C) (Sigma).

### Immunostaining

Neuro-2a cells were cultured on coverslips (Proscitech) within 12-well plates, transfected and treated with 0.3 mM arsenite, as indicated. Subsequent processing was performed in phosphate-buffered saline at room temperature as follows: washing, permeabilization (0.2% (v/v) Triton X-100), fixation (4% (w/v) paraformaldehyde) and blocking (1% (w/v) bovine serum albumin (BSA)). Cells were incubated sequentially with primary antibodies then fluorophore-conjugated secondary antibodies as indicated, each in 1% (w/v) BSA in phosphate-buffered saline (PBS) for 1 h at room temperature: anti-DDX19A (Abcam, ab108462) (1:100), anti-DDX21 (Atlas antibodies, 10528-1-AP) (1:100), anti-DDX42 (Proteintech, HPA023571) (1:100), anti-DDX46 (Atlas antibodies, 16927-1-AP) (1:100), anti-DHX15 (Proteintech, HPA047047) (1:100). Subsequent detection used Alexa Fluor® 568-conjugated secondary antibodies (A-11004, Invitrogen) (1:400). Nuclei were visualized using 4’,6-diamidino-2-phenylindole (DAPI) (Sigma) (1:10,000 in PBS). Processed coverslips were then mounted in water-based Fluoro-Gel (Proscitech) onto glass slides for visualization.

### RNA staining

For detection of RNA the Click-iT RNA Alexa Fluor 594 imaging kit (ThermoFisher, C10330) was used. Cells were co-incubated with 5-ethynyl uridine during the transfection. After 24 h, cells were fixed and washed; incorporated 5-ethynyl uridine was detected according to the manufacturer’s instructions.

### Confocal laser scanning microscopy (CLSM) and image analysis

Fixed samples were visualized by confocal laser scanning microscopy (CLSM) (Leica TCS SP5 with 63X 1.4 Oil Objective). All imaging studies were performed on >3 independent occasions including preliminary optimization steps. The acquired images were quantitatively analyzed using CellProfiler cell image analysis software (version 2.1.1 for Mac) for ataxin-1 NB size (μm^2^; the size of individual NBs as well as the total size of NBs per nucleus calculated by summing the size of all individual NBs within a nucleus) and the fluorescence intensity of nucleus/cytoplasm ratio (Fn/c) for localization of RNA helicases. In the Fn/c analyses, after applying the “CorrectIllumination” function, cytoplasmic areas were defined by subtracting the area stained with DAPI. Where indicated, acquired images were quantitatively analyzed using Fiji for fluorescence intensity of RNA staining within NBs/non-NBs or total nuclear fluorescence intensity (pixels). Quantitation of the RNA fluorescence ratio of NB/non-NB was calculated using the formula: (F_NB_ − F_background_)/(F_non-NB_ − F_background_), in which F_NB_ represents average RNA fluorescence intensity in the NB area, F_non-NB_ represents average RNA fluorescence intensity in the non-NB area in the nucleoplasm and F_Background_ represents average RNA fluorescence intensity in the background of the image.

### Live cell imaging, fluorescence recovery after photobleaching (FRAP) and fluorescence fluctuation spectroscopy (FFS)

At 24 h post-transfection, culture medium was replaced with pre-warmed Phenol-Red-Free DMEM growth medium (Gibco). Cells were kept in an environmental chamber (37 °C, 5% CO_2_) throughout live cell imaging, as well as the FRAP and FFS acquisitions. Image stacks acquired during GFP-ataxin-1 NB fusion events or FRAP analysis were collected using the CLSM Leica TCS SP5 (63X 1.4 Oil Objective). Images stacks acquired during GFP-ataxin-1 NB fusion events and subsequent fluorescence fluctuation analysis were collected using the Zeiss Elyra 780 (63X 1.2 Water Objective).

For FRAP, GFP was excited with the 488 nm emission line of an Argon laser with maximum laser power (50% for Leica TCS SP5) lasting one frame used for photobleaching. Three frames were recorded before photobleaching at a minimal interval (1.3 s). Fluorescence recovery was recorded at 5 s intervals for 250 s. Fluorescence intensity was measured using Fiji. Recovery curve fitting and maximum fluorescence recovery were calculated in SigmaPlot 12.0. Initial rate was calculated as the average fluorescence recovery rate for the first 15 s.

For FFS, 1000 frames (64 × 64 pixel) were acquired using a digital zoom of 10 (13.5 μm region of interest) and a 12.61 μs pixel dwell time which resulted in a line time of 1.89 ms and frame time of 121 ms. GFP was excited with the 488 nm emission line of an Argon laser and detected using the 510–560 nm emission range. The average and variance were calculated in each pixel of every 80 frames (approximately every 10 s per segment) within the 1000 frame acquisition using the Number and Brightness algorithms as executed by the SimFCS software developed at the University of California Irvine Laboratory for Fluorescence Dynamics (www.lfd.uci.edu).

### ATP measurement

Cellular ATP levels were measured using a luminescent ATP detection assay kit (Abcam, ab113849) according to the manufacturer’s instructions.

### Gene knockdown by siRNA

ON-TARGETplus SMARTpool siRNAs were purchased from Dharmacon: D1Pas1(L-059783-01-0005), DDX1(E-052098-00-0005), DDX18(L-063435-01-0005), DDX19A(L-045066-01-0005), DDX21(L-046395-01-0005), DDX24(L-042299-01-0005), DDX3Y(L-043317-01-0005), DDX41(L-052130-00-0005), DDX42(L-054910-01-0005), DDX46(L-066260-01-0005), DHX15(L-044833-01-0005), DHX9(L-056729-02-0005), UPF1(L-050929-01-0005). Each siRNA was co-transfected with GFP-ataxin-1[85Q] using DharmaFECT Duo Transfection Reagent (T-2010-03). Efficacy of knockdown by each siRNA was assessed by cell lysis and Western blot as described in the following section.

### Cell lysis and western blot

Cell lysates were prepared in RIPA buffer [50 mM Tris-HCl, pH 7.3, 150 mM NaCl, 0.1 mM ethylenediaminetetraacetic acid (EDTA), 1% (v/v) sodium deoxycholate, 1% (v/v) Triton X-100, 0.2% (w/v) NaF and 100 µM Na_3_VO_4_] supplemented with Complete protease inhibitor mix (Roche Diagnostic). Cell lysates were incubated on ice (20 min), cleared by centrifugation, and protein concentrations determined using the BioRad protein assay.

Protein samples were separated by SDS-PAGE (8% polyacrylamide gels, 1.5 h) and transferred to polyvinylidene difluoride (PVDF) membranes (Amersham Life Science; 2 h, room temperature). Subsequent steps were performed by blocking with 1% (w/v) BSA in phosphate-buffered saline (PBS; 0.5 h, room temperature), and incubating with primary antibodies (all anti-DDX antibodies as per those used in immunofluorescence analyses) and horseradish peroxidase-linked secondary antibodies (ThermoFisher Scientific) (1:1000) for visualization using an enhanced chemiluminescence detection system (ThermoFisher Scientific). Images were captured using ChemiDoc imager (Bio-Rad) operating in a single-channel protocol.

### Cell death assessment

Neuro-2a cells (2 × 10^5^ cells/well) with transfection as indicated in different conditions, were suspended in PBS and transferred to 5 ml glass round-bottom tubes (BD Bioscience). To determine the numbers of dead cells per sample, the SYTOX Red dead cell stain (ThermoFisher Scientific) was added to cell suspensions, and incubated at room temperature for 15 min before flow cytometry analysis according to the manufacturer’s instructions. All samples were prepared and measured in triplicate, with n = 3 independent experiments performed.

Fluorescence detection and recording was assessed by flow cytometry (BD LSRFortessa (BD Bioscience)) with the background fluorescence control estimated from using untransfected cells and negative controls determined by the GFP-only cells (GFP, Green channel) and SYTOX-only cells (SYTOX, Red channel). The voltage applied to each channel was adjusted according to the GFP-only or SYTOX-only controls to avoid the fluorescence count exceeding 100 K. For analysis, 50,000–75,000 cells were recorded by the cytometer for each sample. FlowJo software (Version vX.0.7) was used in the analysis. The gating of debris and intact Neuro-2a cells was based on forward scatter-height (FSC-H) and the dead cell stain (APC-A). Further gating of single cells was based on the side scatter-height (SSC-H) and side scatter-width (SSC-W) or FSC-H and forward scatter-width (FSC-W). The gating for transfected cells was based on fluorescence GFP-A (GFP, Green channel); the gate for dead cells was based on fluorescence APC-A (SYTOX, Red channel). Cell death percentage (%) in each fluorescence range was determined using the following formula: Number of 100 × (dead cells/number of total cells).

### Statistical analysis

Graphpad Prism 6 (version 6.00 for Mac) was used for statistical analysis. Data are represented as mean ± standard error of the mean (SEM). Data sets were analyzed by D’Agostino-Pearson omnibus normality test before further comparison analysis. For datasets with normal distribution, an unpaired two-tailed student’s t-test (for 2 data sets) or analysis by ANOVA (for > 2 data sets) was applied to compare differences among different datasets, followed by Tukey’s multiple comparisons test; *p < 0.05, **p < 0.01, ***p < 0.001, ****p < 0.0001. For datasets with deviation from normal distribution, Mann-Whitney and Kruskal-Wallis non-parametric test was used for analysis; ^#^p < 0.05, ^##^p < 0.01, ^###^p < 0.001, ^####^p < 0.0001.

## Supplementary information

Supplementary Movie 1.

Supplementary Figures and Legends.

## Data Availability

Materials used and data analysed during the current study are available from the corresponding authors on reasonable request.
