# Supplementary material for: Nuclear bodies formed by polyQ-ataxin-1 protein are liquid RNA/protein droplets with tunable dynamics
Source: Sci Rep. 2020 Jan 31;10:1557. doi: 10.1038/s41598-020-57994-9 (PMC6994494; doi:10.1038/s41598-020-57994-9)
Supplement: Supplementary file 2 — Supplementary Figures and Legends. [file 41598_2020_57994_MOESM2_ESM.pdf]

Nuclear bodies formed by polyQ-ataxin-1 protein are liquid RNA/protein droplets with tunable dynamics

Sunyuan Zhang[1], Elizabeth Hinde[1,2,3], David A. Jans[4], Molly P. Schneider[1], and Marie A. Bogoyevitch[1,*]

[1]Department of Biochemistry and Molecular Biology, [2]Bio21 Molecular Science and Biotechnology Institute, and [3]School of Physics, University of Melbourne, Parkville, Victoria 3010, Australia
[4]Nuclear Signalling Lab., Department of Biochemistry and Molecular Biology, Monash University, Clayton, Victoria 3800, Australia
* corresponding author: marieb@unimelb.edu.au

Supplementary Figure Legends

Supp figure 1. Following photobleaching of GFP-ataxin-1[85Q], fluorescence recovers from both within the ataxin-1 NB and the surrounding nucleoplasm.

Neuro-2a cells were transfected to express GFP-ataxin-1[85Q]. At 24 h post-transfection, cells were used for FRAP. (A) Representative images are shown from 3 independent experiments for quantitative assessments of GFP-ataxin-1[85Q] exchange dynamics. White rectangles (in pre-bleach images) and open arrowheads (in post-bleach images) indicate the ataxin-1 NB analyzed. Different ROIs (ROI 1: across the NB, ROI 2: inside the NB, ROI 3: the entire NB; indicated by white open arrowheads) were photobleached and fluorescence recovery subsequently monitored post-bleach at 5 s intervals for 250 s. Scale bar = 10 μm. (B) Plot of fluorescence recovery (%) from experiments as shown in (A). Each symbol represents fluorescence measured at the indicated time. (C-D) Pooled data from FRAP experiments measuring recovery of fluorescence for GFP-ataxin-1[85Q] over time. Each symbol represents a single data point. The results are also represented as the mean ± SEM for (C) fluorescence maximum recovery percentage in the bleached area (where 100% represents full recovery) and (D) initial rates (average percentage recovery of fluorescence in the first 15 s) (Fn%/s). (Significance values calculated by ANOVA, $n > 10$, *$p<0.05$, **$p<0.01$, ***$p<0.001$, n.s. = not significant).

Supp figure 2: Fusion of ataxin-1[85Q] NBs observed during live cell imaging.

Neuro-2a cells were transfected to express GFP-ataxin-1[85Q]. At 24 h post-transfection, live cell imaging reveals fusion events shown here for two NBs (white square in the top panel, shown as enlarged images in the zoom panels). Representative images are shown from 3 independent experiments. All scale bars = 10 μm.

Supp figure 3: Arsenite treatment increases ataxin-1[85Q] NB average size but does not change the total NB size per nucleus.

Neuro-2a cells were transfected to express GFP-ataxin-1[85Q]. At 24h post-transfection, cells were left untreated (Ctrl) or treated with arsenite (0.3 mM, 1 h) as indicated. Cells were then fixed and stained with DAPI before CLSM imaging. (A) Representative images from 3 independent experiments (~100 cells per condition) for quantitation of NB average size and the total NB size per nucleus are shown. White arrows indicate NBs with visibly increased size upon arsenite exposure. Scale bar = 50 μm. (B-C) Quantitation of NB size. Each symbol represents a single data point. The results are also represented as the mean ± SEM for (B) average size of ataxin-1 NBs or the (C) total size of the ataxin-1 NBs per nucleus. (Significance values calculated by unpaired two-tailed student's t-test, *$p<0.05$, n.s. = not significant).

Supp figure 4: Ataxin-1[85Q] self association domain mutant (ΔSAD) forms smaller NBs that do not increase in size following arsenite stress.

Neuro-2a cells were transfected to express GFP-ataxin-1[85Q] or GFP-ataxin-1[85Q]ΔSAD. At 24h post-transfection, cells were left untreated or treated with arsenite (0.3 mM, 1 h) as indicated. Cells were then fixed and stained with DAPI before CLSM imaging. (A) Representative images from 3 independent experiments (~100 cells per condition) for quantitation of NB average size are shown. White squares in the right panels are shown as enlarged images in the zoom panels. Scale bars = 50 μm (left panels without zoom) or 10 μm (right panels with zoom). (B) Quantitation of NB size. Each symbol represents a single data point. The results are also represented as the mean ± SEM. (Significance values calculated by ANOVA, ***p<0.001, n.s. = not significant). (C) Cell death percentage for the transfected cell population was assessed by staining with the SYTOX Red dead cell stain followed by flow cytometry analysis.

Supp figure 5: Ataxin-1[30Q] also forms NBs that show high dynamics and fusion

Neuro-2a cells were transfected to express GFP-ataxin-1[85Q]. At 24h post-transfection, cells were incubated in an imaging chamber equilibrated with 5% CO_2 at 37°C prior to FRAP using CLSM. (A) Representative images are shown from 3 independent experiments for quantitative assessments of GFP-ataxin-1[85Q] exchange dynamics. White rectangle indicates ataxin-1 NB analyzed. A small ROI (indicated by white open arrowhead for dynamic NB) was photobleached and fluorescence recovery was subsequently monitored post-bleach at 5 s intervals for 250 s. (B) Plot of fluorescence recovery (%) from experiments as shown in (A). Each symbol represents fluorescence measured at the indicated time. (C) Pooled data from FRAP experiments measuring recovery of fluorescence for GFP-ataxin-1[30Q] over time (average percentage recovery of fluorescence in the first 15 s) (Fn%/s). Each symbol represents a single data point. The results are also represented as the mean ± SEM (n > 7). (D) Live cell imaging reveals fusion events shown here for two NBs (white square in the top panel, shown as enlarged images in the zoom panels). Representative images are shown from 3 independent experiments. All scale bars = 10 μm.

Supp figure 6: Arsenite or sorbitol stress treatment does not alter total GFP-ataxin-1[85Q] fluorescence intensity.

Neuro-2a cells were transfected to express GFP-ataxin-1[85Q]. At 24h post-transfection, cells were left untreated or treated with arsenite (0.3 mM, 1 h) or sorbitol (0.5M, 1h) as indicated. Cells were then fixed and stained with DAPI before CLSM imaging using identical settings and laser intensity for all three conditions. (A) Representative images from 3 independent experiments (~100 cells per condition) for quantitation of total GFP-ataxin-1[85Q] fluorescence intensity per nucleus are shown. Scale bars = 50 μm. (B) Quantitation of total GFP-ataxin-1[85Q] fluorescence intensity per nucleus. Each symbol represents a single data point. The results are also represented as the mean ± SEM. (Significance values calculated by ANOVA, ***p<0.001, n.s. = not significant).

Supp figure 7: Intracellular ATP is depleted by CCCP+2DG treatment but recovers after inhibitor wash-out.

Neuro-2a cells were transfected to express GFP-ataxin-1[85Q] in a 96-well plate. At 24 h post-transfection, cells were treated for the times indicated with 2DG and CCCP (inhibitors of glycolysis and oxidative phosphorylation, respectively). For ATP recovery, inhibitors were removed by three washes with recovery for the times indicated. ATP levels were measured using a luminescent detection assay kit. (Significance values calculated by ANOVA, n=3 independent experiments, **p<0.01, ****p<0.0001, n.s. = not significant).

Supp figure 8: siRNA-mediated knockdown of RNA helicases.

Neuro-2a cells were co-transfected with GFP-ataxin-1[85Q] and different siRNAs: negative ctrl siRNA, siDDX21, siDDX19A, siDDX42, siDDX46, or siDHX15. Protein lysates were analysed by Western blotting using specific antibodies to the following proteins: DDX21, DDX19A, DDX42, DDX46 and DHX15; detection of tubulin was used as a loading control. Arrows indicate the protein bands of interest according to the size predicted for each protein. Representative results of full-length blots are shown from 3 independent experiments.

Supp figure 9: Endogenous RNA helicases DDX42, DDX46 and DHX15 show nuclear localization.

Neuro-2a cells were transfected to express GFP-ataxin-1[85Q]. At 24 h post-transfection, cells were left untreated or treated with arsenite (0.3 mM, 1 h) as indicated. (A) Cells were then fixed and immunostained to visualize the RNA helicases DDX21, DHX19A, DDX42, DDX46 and DHX15, together with DAPI staining of DNA, before CLSM imaging. All scale bars = 10 μm. Representative images are shown from 3 independent experiments. (B) Quantitative analysis for the fluorescence ratio nucleus to cytoplasm (Fn/c) for each of the RNA helicases detected with immunostaining. Each symbol represents a single data point. The results are also represented as the mean ± SEM (Significance values calculated by ANOVA, n>10, *p<0.05, ****p<0.0001, n.s. = not significant).

Supp figure 10: siRNA-mediated knockdown of RNA helicases DDX42, DDX46, or DHX15 does not increase cell death.

Neuro-2a cells were co-transfected with GFP and different siRNAs: negative ctrl siRNA, siDDX42, siDDX46, or siDHX15. At 24 h post-transfection, cells were collected into round-bottom glass tubes, and added with SYTOX Red dead cell stain for 15 min prior to fluorescence detection by flow cytometry (BD LSRFortessa). (A) Representative gates are shown for (I) forward scattering and APC-A (SYTOX Red) gates to focus on the intact cell population, (II) side scattering by height (SSC-H) and width (SSC-W) focusing on the single cell population, (III) forward scattering by height (FSC-H) and width (FSC-W) focusing on the single cell population. (B) Representative images are shown for gates applied to each siRNA condition (Neg ctrl, siDDX42, siDDX46, siDHX15) with GFP transfected cells (GFP-A positive) or non-transfected cells (NT) (GFP-A negative), and dead cells (APC-A positive) or live cells (APC-A negative). (C) Cell death percentage among transfected cells in all conditions. Results are calculated from 3 independent experiments. (Significance values calculated by ANOVA, *p<0.05, ***p<0.001, ****p<0.0001, n.s. = not significant).

Supp movie 1: Fusion of ataxin-1[85Q] NBs visualized during live cell imaging.

Neuro-2a cells were transfected to express GFP-ataxin-1[85Q]. At 24 h post-transfection, images were recorded for 5 min (60 frames at 5 s intervals). Image stacks were compressed into a video (0.2 s per frame).

Supp Fig 1

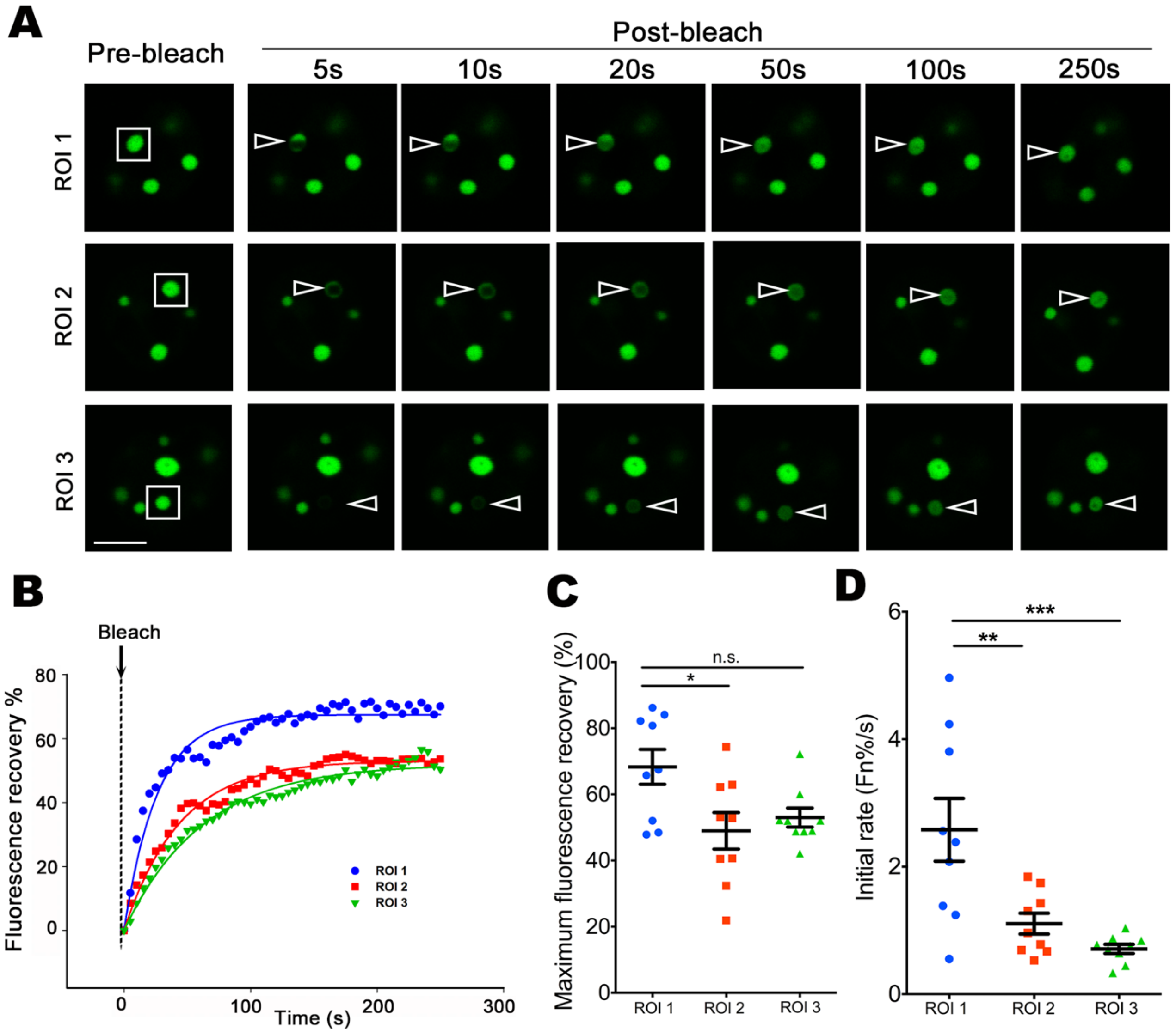

Supp Fig 2

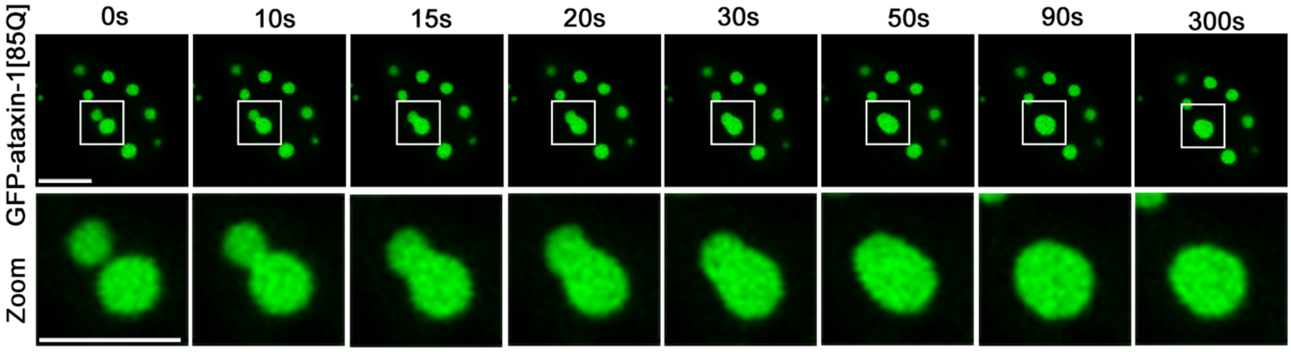

Supp Fig 3

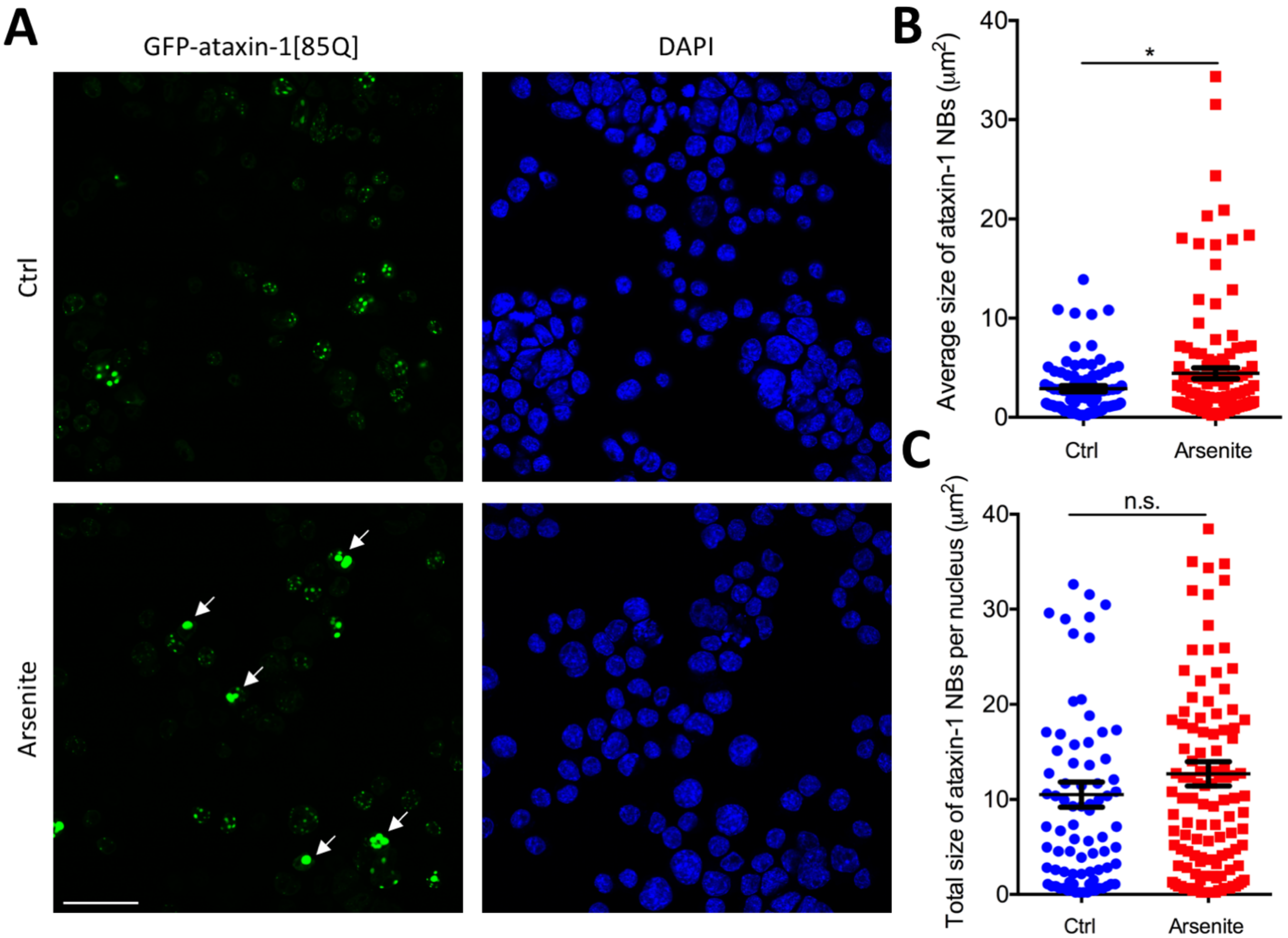
A
GFP-ataxin-1[85Q]
DAPI
Ctrl
Arsenite
B
Average size of ataxin-1 NBs (μm2)
*
40
30
20
10
0
Ctrl
Arsenite
C
Total size of ataxin-1 NBs per nucleus (μm2)
n.s.
40
30
20
10
0
Ctrl
Arsenite

Supp Fig 4

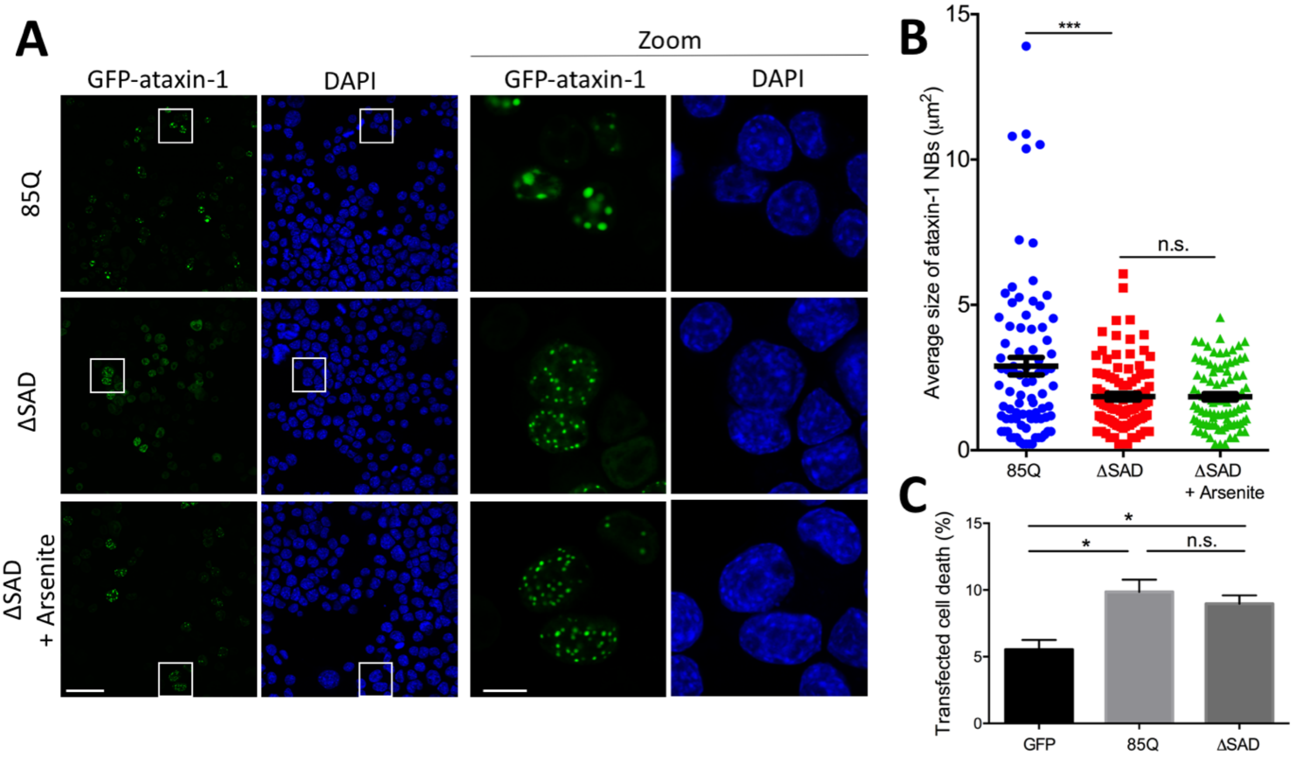

A
Zoom
GFP-ataxin-1
DAPI
GFP-ataxin-1
DAPI
85Q
ΔSAD
ΔSAD + Arsenite
B
***
n.s.
Average size of ataxin-1 NBs (μm2)
15
10
5
0
85Q
ΔSAD
ΔSAD + Arsenite
C
*
*
n.s.
Transfected cell death (%)
15
10
5
0
GFP
85Q
ΔSAD

Supp Fig 5

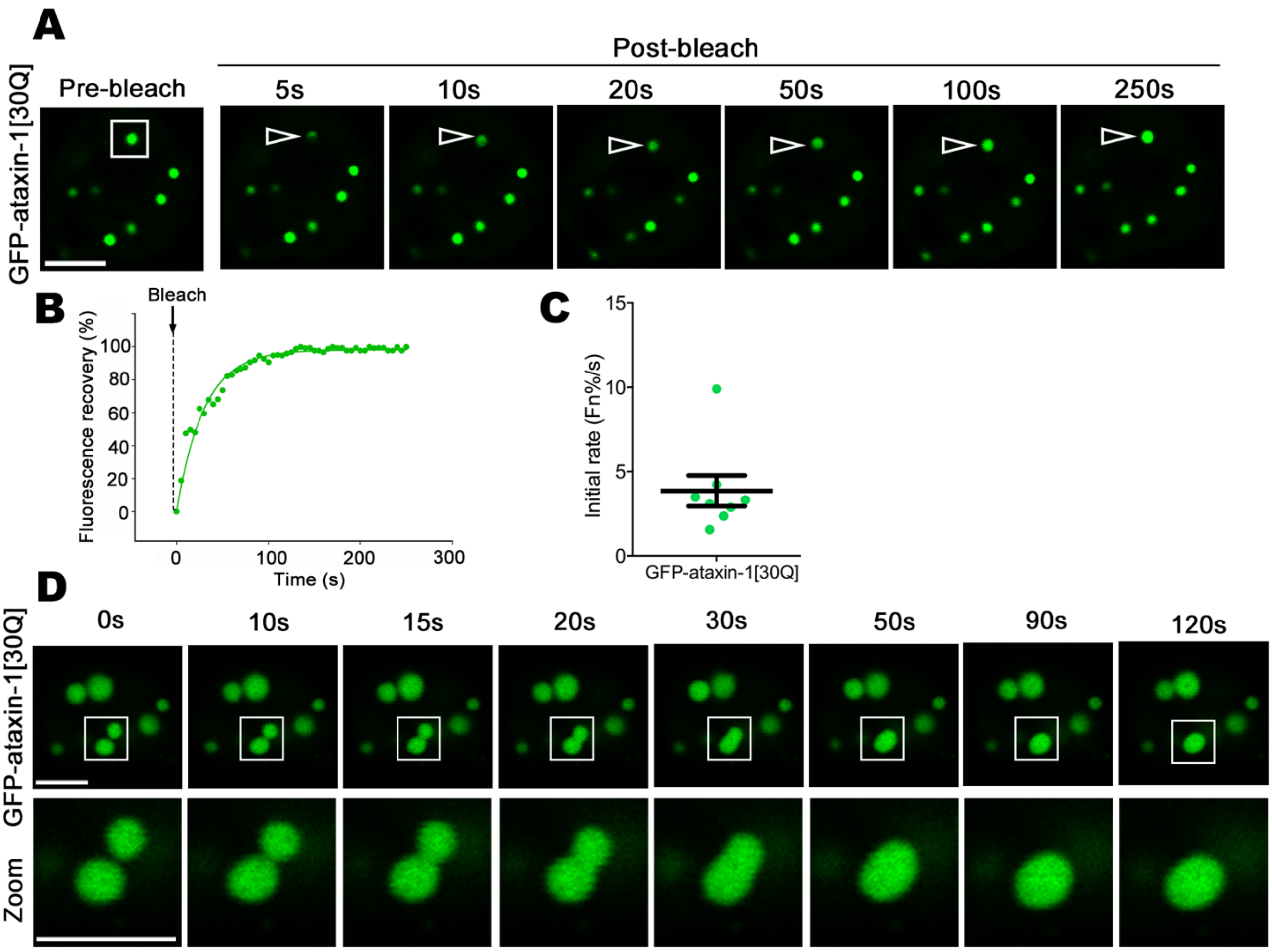
A
Post-bleach
GFP-ataxin-1[30Q]
Pre-bleach
5s
10s
20s
50s
100s
250s
B
Bleach
Fluorescence recovery (%)
0
20
40
60
80
100
0
100
200
300
Time (s)
C
Initial rate (Fn%/s)
0
5
10
15
GFP-ataxin-1[30Q]
D
0s
10s
15s
20s
30s
50s
90s
120s
GFP-ataxin-1[30Q]
Zoom

Supp Fig 6

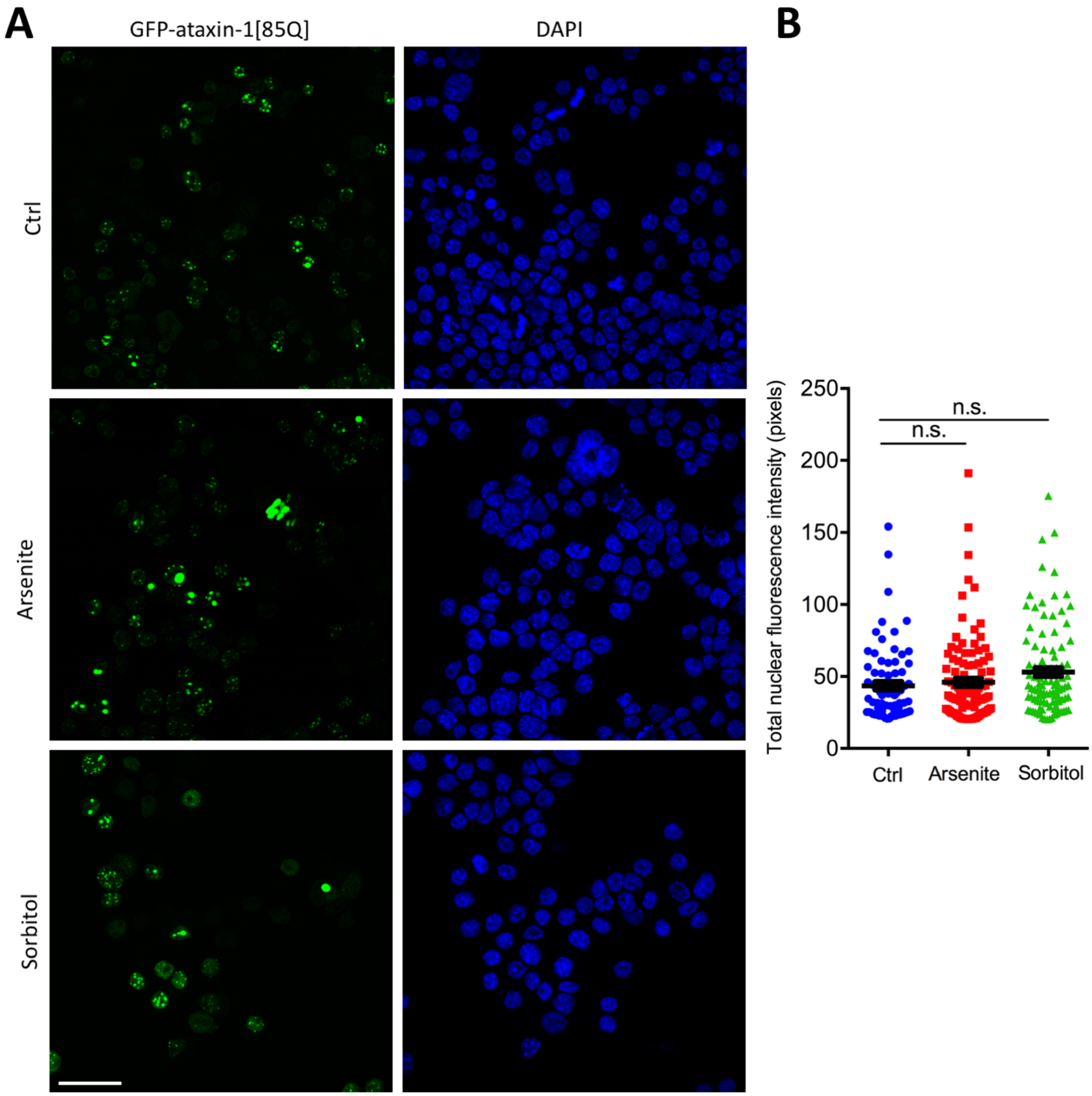
A
GFP-ataxin-1[85Q]
DAPI
Ctrl
Arsenite
Sorbitol
B
Total nuclear fluorescence intensity (pixels)
250
200
150
100
50
0
n.s.
n.s.
Ctrl
Arsenite
Sorbitol

Supp Fig 7

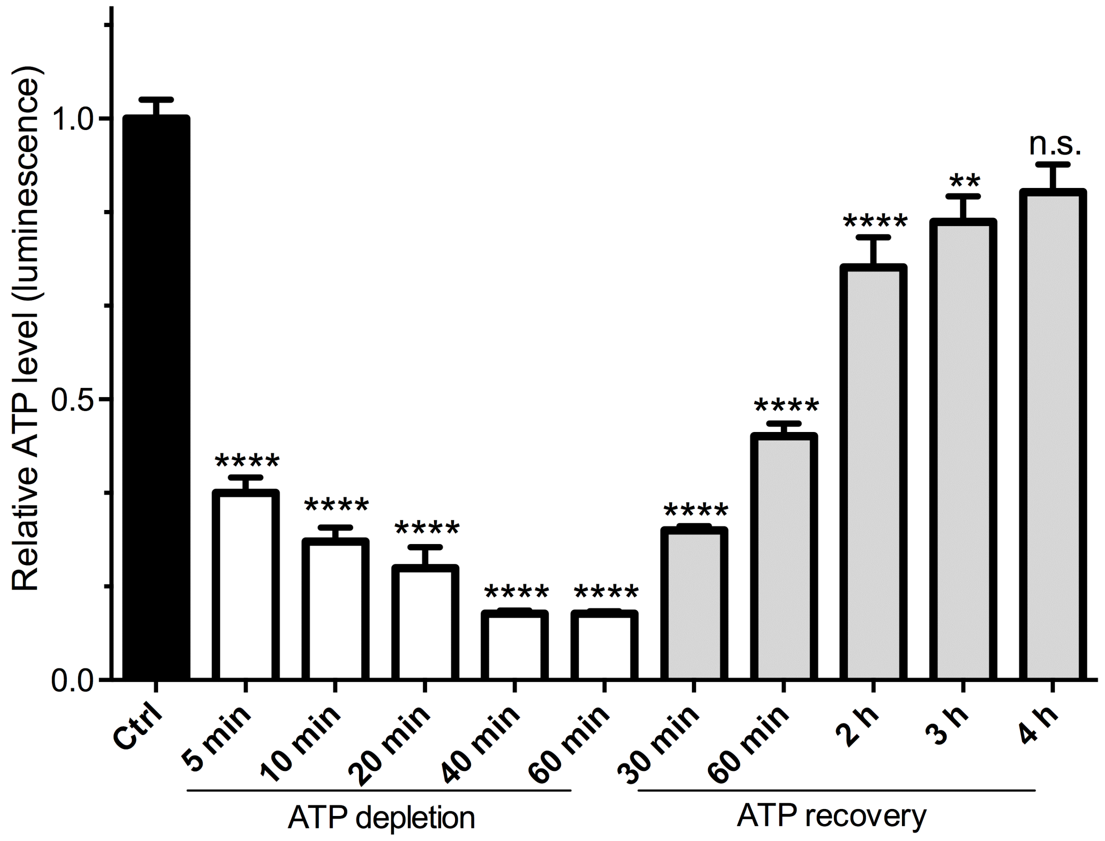
Relative ATP level (luminescence)
1.0
0.5
0.0
****
****
****
****
****
****
****
****
**
n.s.
Ctrl
5 min
10 min
20 min
40 min
60 min
30 min
60 min
2 h
3 h
4 h
ATP depletion
ATP recovery

Supp Fig 8

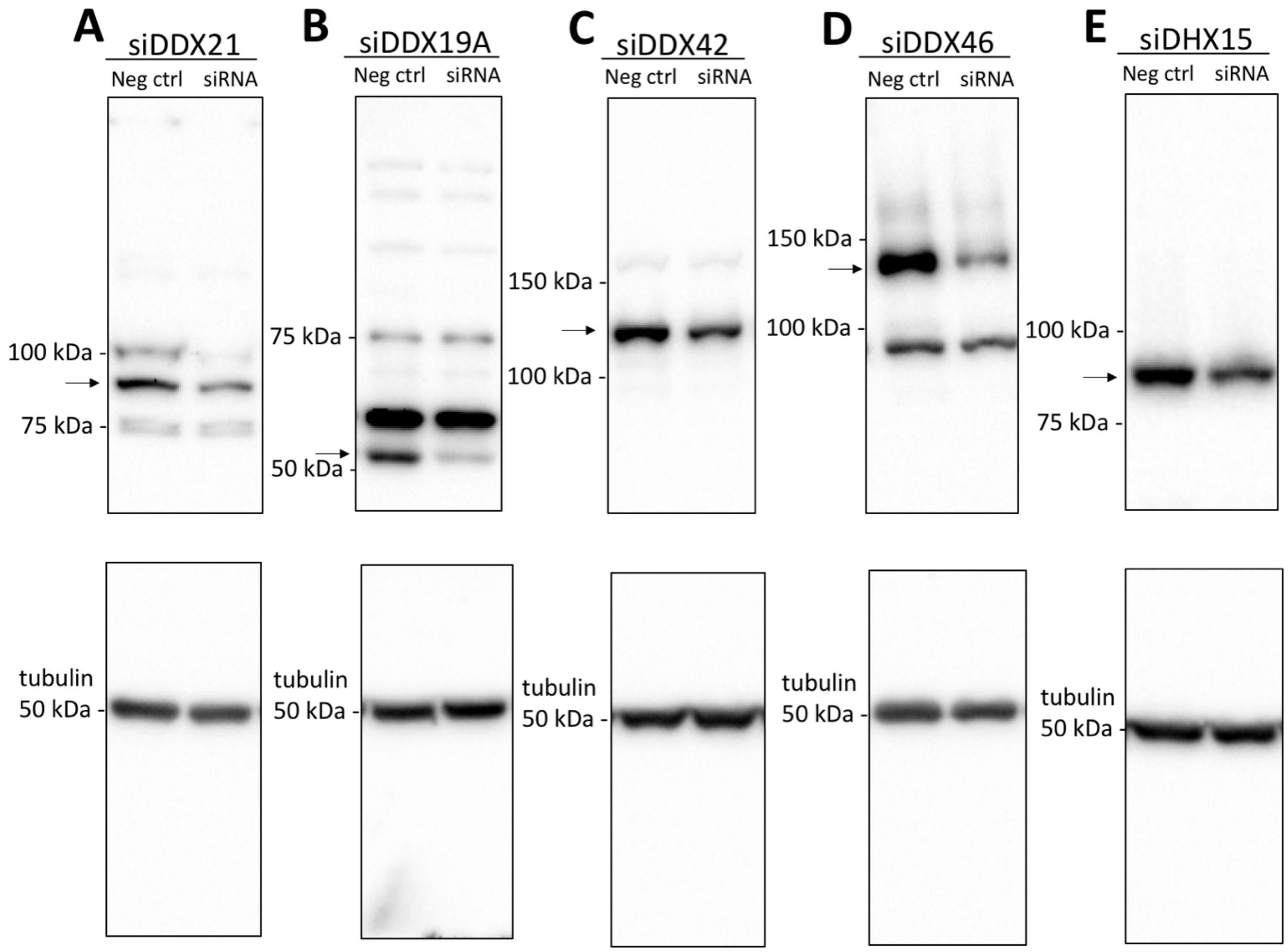
A
siDDX21
Neg ctrl
siRNA
100 kDa
75 kDa
tubulin
50 kDa
B
siDDX19A
Neg ctrl
siRNA
75 kDa
50 kDa
tubulin
50 kDa
C
siDDX42
Neg ctrl
siRNA
150 kDa
100 kDa
tubulin
50 kDa
D
siDDX46
Neg ctrl
siRNA
150 kDa
100 kDa
tubulin
50 kDa
E
siDHX15
Neg ctrl
siRNA
100 kDa
75 kDa
tubulin
50 kDa

Supp Fig 9

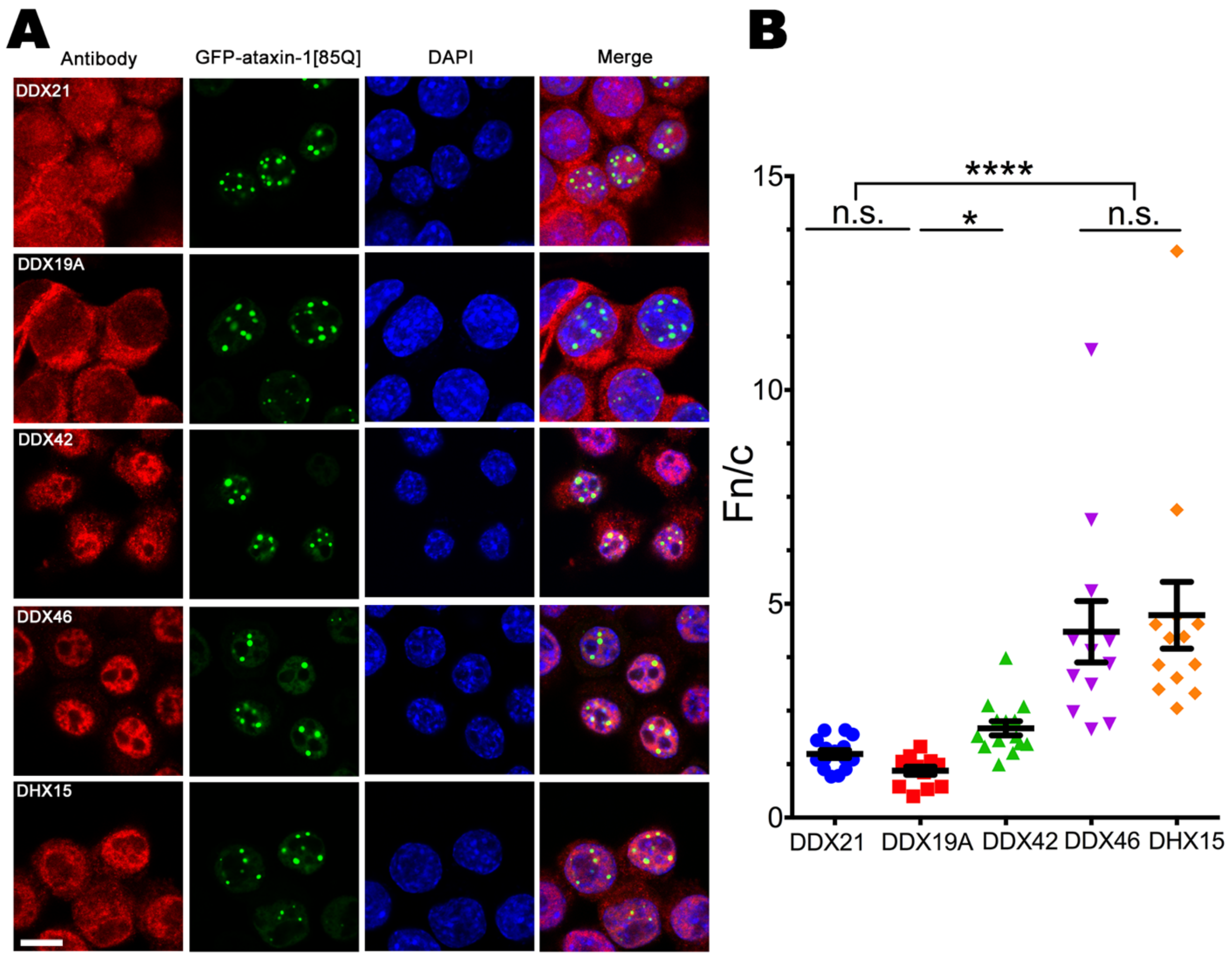
A
Antibody
GFP-ataxin-1[85Q]
DAPI
Merge
DDX21
DDX19A
DDX42
DDX46
DHX15
B
Fn/c
15
10
5
0
****
n.s.
*
n.s.
DDX21
DDX19A
DDX42
DDX46
DHX15

Supp Fig 10

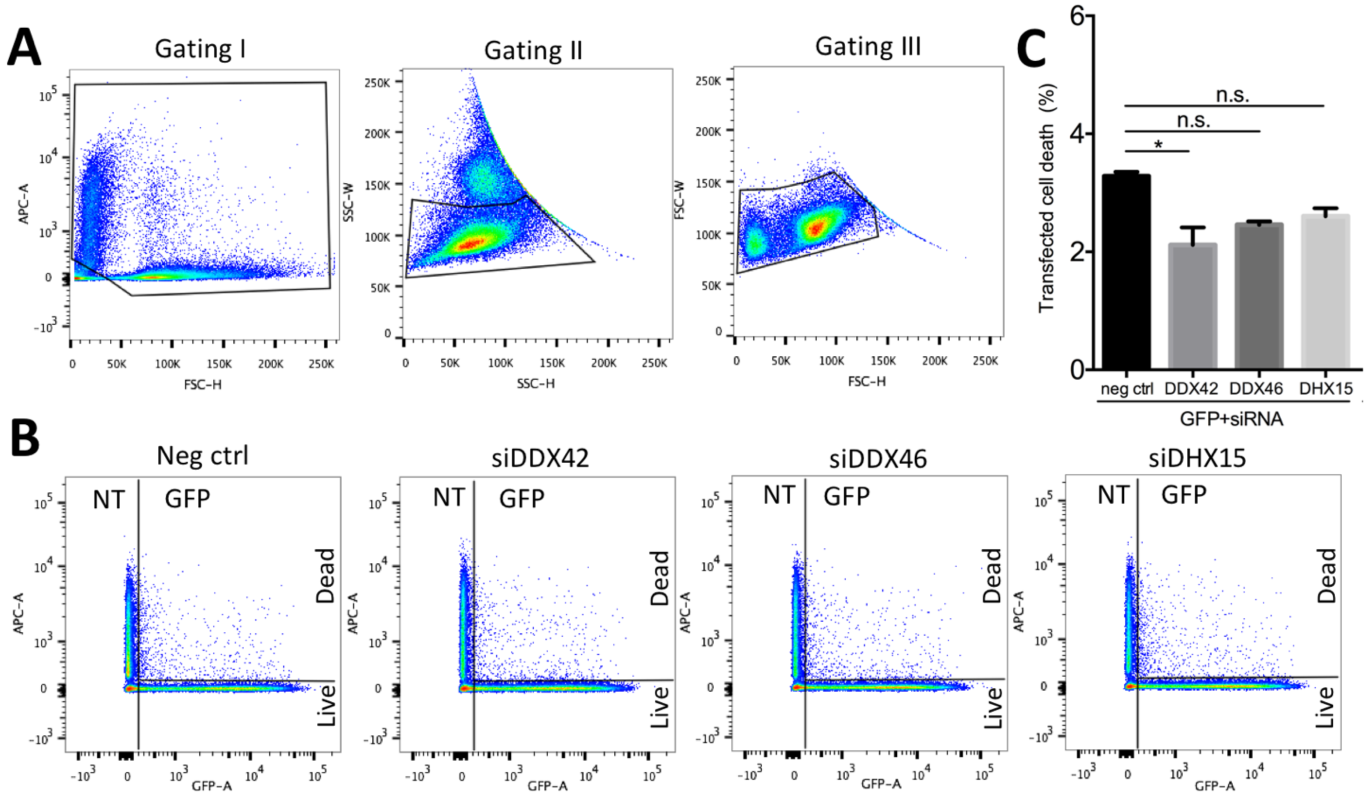
A
Gating I
Gating II
Gating III
APC-A
FSC-H
SSC-W
SSC-H
FSC-W
C
Transfected cell death (%)
n.s.
n.s.
*
neg ctrl
DDX42
DDX46
DHX15
GFP+siRNA
B
Neg ctrl
siDDX42
siDDX46
siDHX15
NT
GFP
Dead
Live
GFP-A
